# Cortical inhibitory but not excitatory synaptic transmission and circuit refinement are altered after the deletion of NMDA receptors during early development

**DOI:** 10.1038/s41598-023-27536-0

**Published:** 2023-01-12

**Authors:** Rongkang Deng, Minzi Chang, Joseph P. Y. Kao, Patrick O. Kanold

**Affiliations:** 1 Department of Biomedical Engineering, School of Medicine, Johns Hopkins University, 733 N. Broadway Avenue / Miller 379, Baltimore, MD 21205 USA; 2 Department of Biology, University of Maryland, College Park, MD 20742 USA; 3 Biological Sciences Graduate Program, University of Maryland, College Park, MD 20742 USA; 4 Center for Biomedical Engineering and Technology, Department of Physiology, University of Maryland School of Medicine, Baltimore, MD 21201 USA

**Keywords:** Neural circuits, Neuronal development, Synaptic development, Cortex

## Abstract

Neurons in the cerebral cortex form excitatory and inhibitory circuits with specific laminar locations. The mechanisms underlying the development of these spatially specific circuits is not fully understood. To test if postsynaptic *N*-methyl-D-aspartate (NMDA) receptors on excitatory neurons are required for the development of specific circuits to these neurons, we genetically ablated NMDA receptors from a subset of excitatory neurons in the temporal association cortex (TeA) through in utero electroporation and assessed the intracortical circuits connecting to L5 neurons through in vitro whole-cell patch clamp recordings coupled with laser-scanning photostimulation (LSPS). In NMDAR knockout neurons, α-amino-3-hydroxy-5-methyl-4-isoxazolepropionic acid (AMPA) receptor-mediated connections were largely intact. In contrast both LSPS and mini-IPSC recordings revealed that γ-aminobutyric acid type A (GABA_A_) receptor-mediated connections were impaired in NMDAR knockout neurons. These results suggest that postsynaptic NMDA receptors are important for the development of GABAergic circuits.

## Introduction

Neurons form intricate and highly specific connections that are important for neural computation. For example, neurons in the neocortex form canonical circuits that are thought to underlie higher cognitive functions performed by the cortex^1,2^. Sensory information processing involves sensorily evoked responses in both primary cortices as well as in higher order areas such as the temporal association area (TeA)^3–6^. In particular, TeA is in close proximity to the primary auditory cortex (A1), receives dominant inputs from A1, and is involved in auditorily driven plasticity^7,8^. Abnormal connections in the TeA during early development is a potential substrate of neurological disorder such as autism spectrum disorders^9,10^. Yet the synaptic development in TeA has not been well studied.

NMDA receptors are critically involved in normal cortical development^11–13^. Unlike mature neurons, in which ionotropic glutamatergic transmission is mediated by both AMPA and NMDA receptors (AMPAR and NMDAR), immature neurons have a higher level of NMDARs relative to AMPARs^14–17^ or lack AMPARs completely^18–25^. The NMDAR-only connections are thought to be ‘silent’, because activation of NMDARs requires membrane depolarization to relieve magnesium blockade of NMDAR channels^26–28^. During development, AMPARs gradually increase and ‘unsilence’ the NMDAR-only connections and this process is thought to be mediated by the coordinated action of excitatory GABAergic synaptic transmission and activation of NMDARs in the immature neurons^29–31^. Moreover, because activation of NMDARs and their downstream signaling is part of the mechanism for recruiting AMPARs into the postsynaptic sites during long-term potentiation^32^, NMDARs may play a similar role in shaping the development of AMPAR circuits^18,20^.

Fast inhibitory synaptic communication in the cortex is mediated by ionotropic GABA_A_ receptors (GABA_A_R) and malfunctioning of GABAergic synaptic transmission is thought to underlie various neurological disorders^33,34^. Thus, the mechanisms regulating the development of GABAergic synaptic connections are important. NMDARs are crucial for the development of GABAergic synaptic connections^35–37^. Genetic deletion of NMDARs leads to reduction of GABAergic synaptic connections that depends on the calcium signaling through the NMDARs^38–40^. This evidence suggests that NMDAR signaling is indispensable for the development of GABAergic synaptic connections. Cortical neurons are embedded in microcircuits receiving glutamatergic and GABAergic inputs from specific presynaptic neurons located in specific layers^1^, and these circuits emerge over development^23,25,41–44^. To date, it is unknown if NMDAR signaling in postsynaptic cells contributes to the establishment of the specific laminar microcircuits impinging on them.

As disrupted NMDAR functions and abnormal neural circuits in the temporal cortex are both involved in autism spectrum disorders^9,10,45^, we hypothesized that disrupting NMDAR functions could hinder the development of the glutamatergic and GABAergic circuits onto neurons in TeA. We here tested this hypothesis directly by creating a mosaic cortical deletion of the obligatory NMDAR1 (GluN1) subunit of the NMDA receptors in subsets of pyramidal neurons in TeA through in utero electroporation of Cre plasmid into the cerebral cortex of *Grin1*^*flox*^ mice. We subsequently investigated the development of AMPAR- and GABA_A_R-mediated connections through in vitro electrophysiology in brain slices. We found that NMDARs are not required for the development of action potential-evoked and spontaneous synaptic transmission and laminar circuits mediated by AMPARs. In contrast, we observed a decrease in synaptic transmission mediated by GABA_A_Rs. Thus, postsynaptic NMDARs are required for the normal development of inhibitory but not excitatory synaptic transmission and circuits onto pyramidal cells.

## Results

Here, we set out to test if NMDARs are required for the maturation of spatially specific AMPAR- and GABA_A_R-mediated connections in TeA. We focused on early-developing excitatory neurons in layer 5 (L5).

## Development of AMPAR-mediated connections onto layer 5 excitatory neurons

**Figure 1:**
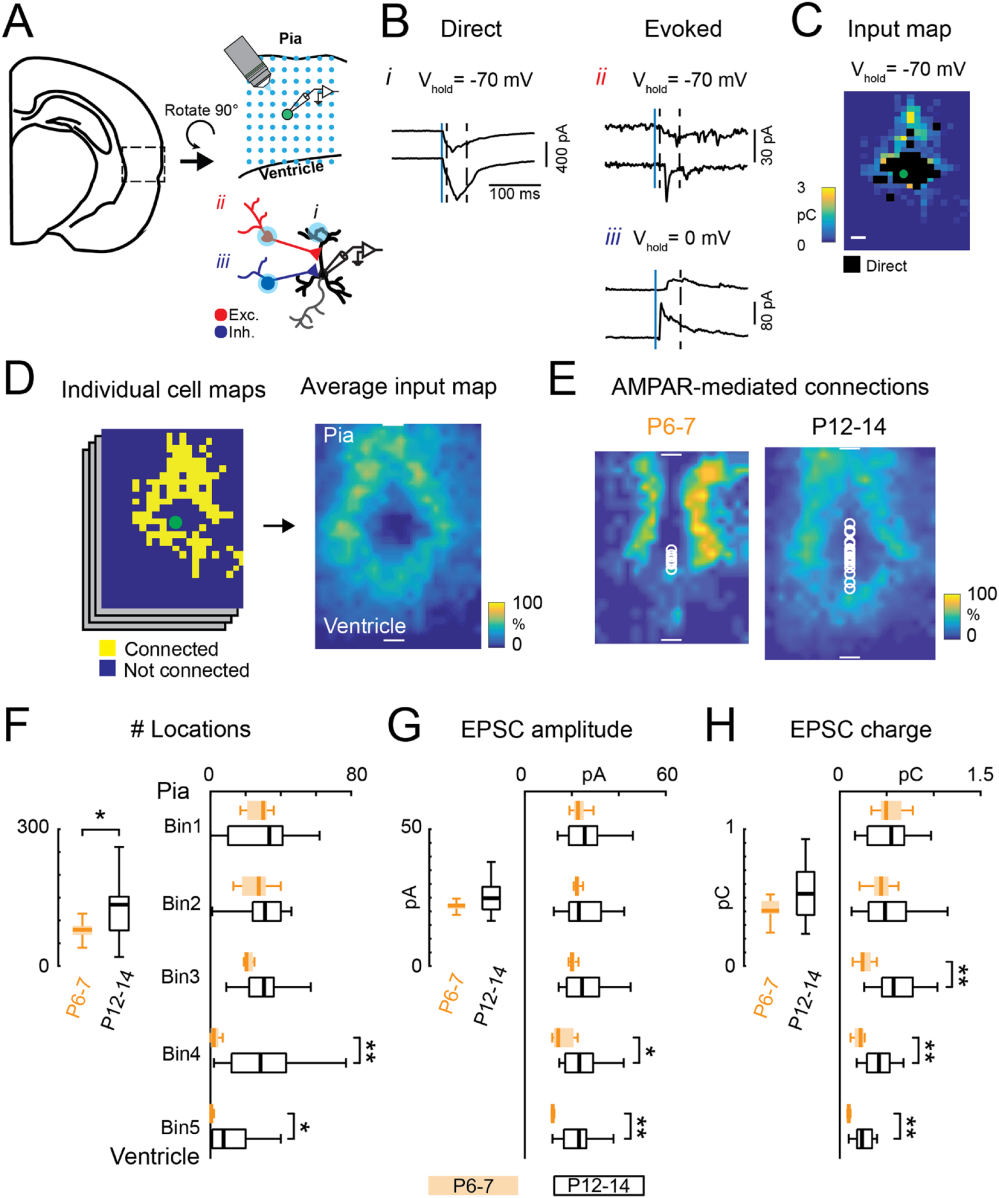
Postnatal development of AMPAR-mediated circuits in L5 excitatory neurons in TeA. (**A**) Illustration of LSPS photostimulation and whole-cell patch clamp recording from excitatory neurons in L5 of TeA. Blue dots indicate that multiple stimulation sites spanning the cortical are activated via glutamate uncaging for each recorded neuron. LSPS directly on the proximal dendrites of the recorded neuron (*i*), and on a presynaptically connected excitatory (*ii*) or inhibitory (*iii*) neuron are shown schematically. (**B**) Exemplar LSPS traces showing direct stimulation of the dendrites of the recorded neuron (*i*), EPSCs evoked by stimulation of presynaptically connected excitatory neurons (*ii*), or IPSCs evoked by stimulation of presynaptically connected GABAergic neurons (*iii*). Blue vertical line indicates the time of LSPS. Dashed vertical lines delimit the time window for capturing LSPS-evoked PSCs (see methods for details). (**C**) Exemplar AMPAR-mediated connection map for a L5 excitatory neuron recorded at a holding potential of -70 mV. Connected locations are color-coded based on the EPSC transferred charge. Black indicates locations omitted in the analysis due to direct LSPS stimulation of the soma or proximal dendrites of the recorded neuron (location *i* in **A**). The green dot marks the soma location of the recorded neuron. Length of white bar represents 100 µm. (**D**) Maps of evoked responses from multiple neurons are averaged to generate the average connection map, which shows the fraction of cells that have synaptic inputs from the different cortical locations. Only evoked responses are used for constructing connection maps and further analysis. White bars mark the average location of pia and ventricle in each slice, and represent 100 µm. (**E**) Average AMPAR-mediated connection maps (maps of evoked EPSC) at P6-7 and P12-14. White circles mark the soma locations for each recorded neuron. Pseudocolor encodes the fraction of cells that have inputs from the different cortical locations. Map at P12-14 is larger due to brain growth. (**F**) Left, total number of effective stimulus locations for AMPAR-mediated connections during postnatal development. Left: P6-7 vs P12-14, *P* = 0.037. Right, laminar distribution of the connected locations. Right: Bin4: P6-7 vs P12-14, *P* = 0.0002. Bin5: P6-7 vs P12-14, *P* = 0.013. (**G**) Left, average EPSC amplitude during development. Right, average EPSC amplitude in different laminar locations. Right: Bin4: P6-7 vs P12-14, *P* = 0.019. Bin5: P6-7 vs P12-14, *P* = 0.005. (**H):** Left, average EPSC transferred charge during development. Right, average EPSC transferred charge in different laminar locations. Right: Bin3: P6-7 vs P12-14, *P* = 0.0002. Bin4: P6-7 vs P12-14, *P* = 0.007. Bin5: P6-7 vs P12-14, *P* = 0.008. F to H, data are presented as box plots. P6-7, n = 8 cells; P12-14, n = 22 cells. (*) *P* < 0.05, (**) *P* < 0.01, otherwise *P* > 0.05. Wilcoxon rank sum test. (**D-H**) data are from electroporated WT animals that were subject to the same electroporation procedures.

Cortical circuits undergo extensive changes during the first few postnatal weeks in mice, including an increase of AMPAR-mediated connections and maturation of the specific laminar connections^23,25,41,43,44,46–48^. To verify that neurons in TeA followed a trajectory similar to primary areas^44^, we first investigated the postnatal development of AMPAR-mediated connections onto L5 excitatory neurons. To identify the sources of functional excitatory and inhibitory inputs we combined whole-cell voltage clamp recording in brain slices with laser-scanning photostimulation (LSPS) to activate presynaptic neurons (Fig. 1A and B). AMPAR-mediated currents were recorded by holding cells at a resting membrane potential of −70 mV. When LSPS released glutamate near the soma of the recorded neuron, glutamate receptors on the recorded cells could be directly activated and the responses can mask the monosynaptically evoked EPSCs we want to study. We distinguished direct activation of the recorded cell from monosynaptically evoked EPSCs by the latency of the evoked currents.The identified monosynaptically evoked EPSCs were sensitive to TTX^25,41,44,49^ (Fig. 1B). For each recorded neuron, we constructed a connection map showing stimulus locations that gave rise to an evoked EPSC (Fig. 1C). To identify the spatial pattern of connections over the population of recorded cells, we aligned the individual connection maps to the pia and ventricle locations and computed the fraction of cells that received inputs from each location, resulting in a spatial probability map or average input map (Fig. 1D). The average input maps for cells recorded at P6-7 and P12-14 showed that over development L5 excitatory neurons received more excitatory connections from locations further away from the recorded cells (Fig. 1E). Quantification of the numbers of inputs of each neuron indicated a significant increase in the total number of connected locations at the end of the second postnatal week (P12-14) (Fig. 1F left). To identify which laminar location contributed to the developmental change, we separated the cortex into 5 equal bins along the laminar direction and calculated the number of connected locations in each laminar group. The analysis showed that the developmental increase of AMPAR-mediated connections observed during the second postnatal week came largely from the lower layers (Fig. 1F right). Accordingly, the peak amplitude and transferred charge also increased for connections coming from lower layers (Fig. 1G and H). Additionally, the spiking responses of cortical neurons to our stimulation protocol are largely constant during postnatal development^44^, suggesting that the observed changes in the average maps between P6-7 and P12-14 reflect functional circuit changes, not changes in the electrical properties of single neurons. Together, the results suggest a developmental increase of AMPAR-mediated connections onto L5 excitatory neurons.

### Layer 5 neurons have NMDAR-only synapses at P7

**Figure 2:**
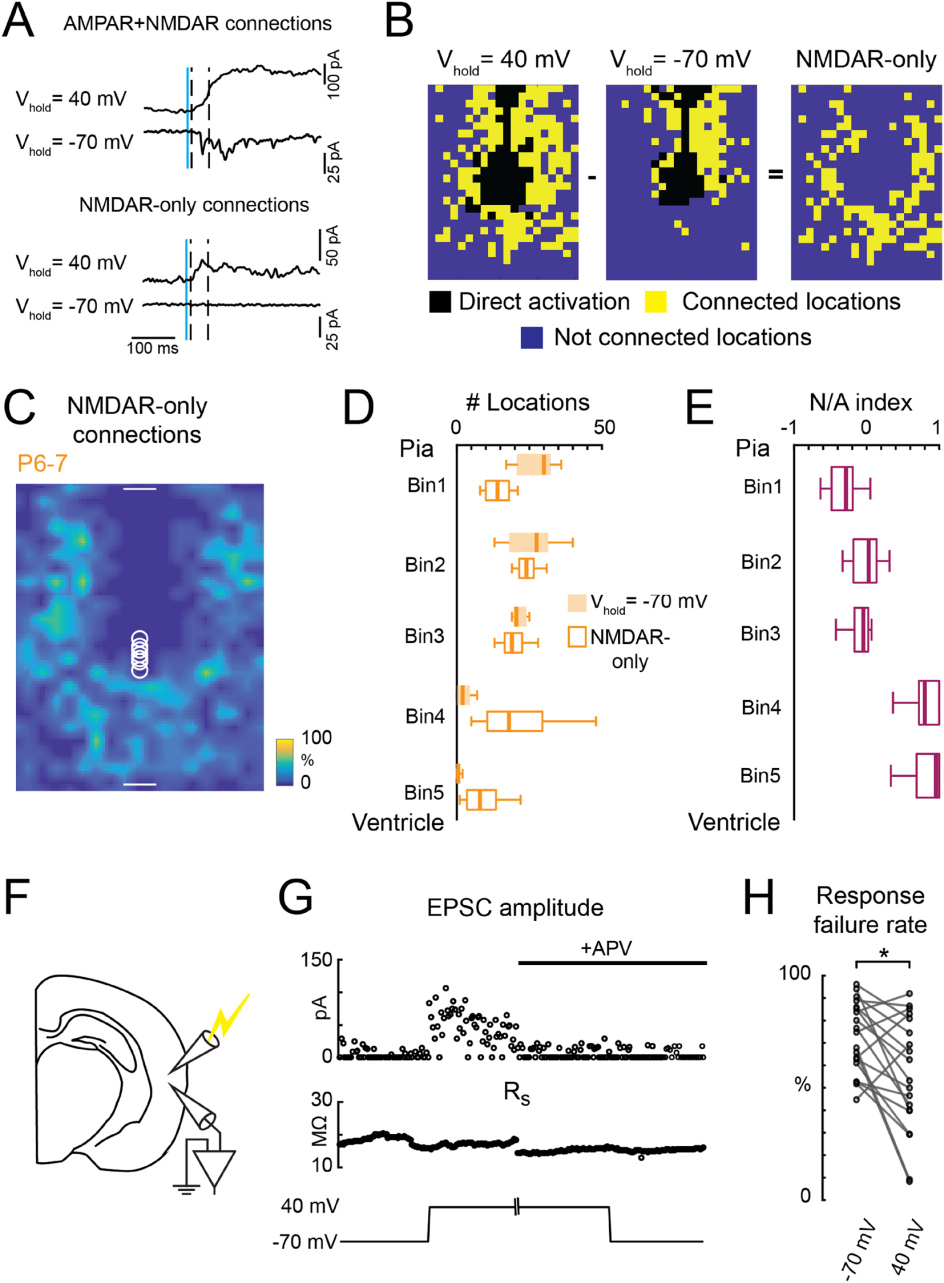
Developing L5 neurons have NMDAR-only connections. (**A**) Identifying NMDAR-only connections through LSPS. Exemplar EPSC traces for connections with both AMPARs and NMDARs (top) and for NMDAR-only connections (bottom). Blue vertical line indicates the time of LSPS. Dashed vertical lines delimit the time window for capturing evoked PSCs. GABA_A_R-mediated currents are blocked by PTX. (**B**) Identification of NMDAR-only connections through LSPS. For each cell, connections were investigated at both -70 mV (middle) and + 40 mV (left) holding potentials. AMPAR-containing connections are revealed at V_hold_ = -70 mV (middle); NMDAR-only connections are revealed at V_hold_ =  + 40 mV (right). Black: locations omitted in the analysis due to direct activation of cells. Yellow: connected locations. Blue: not connected locations. (**C**) Average connection map of NMDAR-only connections onto L5 neurons (n = 8 cells) at P6-7. Color indicates the fraction of cells that have synaptic connections from the different cortical locations. (**D**) Laminar distribution of the connected locations via AMPAR-mediated and NMDAR-only connections. Data for AMPAR-mediated connections are from Fig. 1. (**E**) NMDAR-only/AMPAR-mediated connections ratio (N/A index) in different laminar locations showing that NMDAR-only connections have bigger contributions in lower layers (Bin 4 and Bin 5). **D** and **E**, data are presented as box plots. Bin1 vs Bin4: *P* = 0.0003; Bin1 vs Bin5: *P* = 0.0002. Bin2 vs Bin4: *P* = 0.038. Bin2 vs Bin5: *P* = 0.026. Bin3 vs Bin4: *P* = 0.025; Bin3 vs Bin5: *P* = 0.017. n = 8 cells. Kruskal–Wallis test was used and followed by Tukey's honest significant difference criterion for multi-group comparison. (**F**) Illustration of using minimal electrical stimulation and whole-cell voltage clamp recording to reveal NMDAR-only connections. (**G**) An example cell from the minimal electrical stimulation experiment. Switching V_hold_ to + 40 mV decreased response failure rate (from 0.68 to 0.09). Additional EPSCs at V_hold_ of + 40 mV can be blocked by D-APV. Failure rate went back to 0.58. The absolute amplitude of recorded EPSCs were plotted in the upper panel. Serial resistance (R_s_) is monitored to ensure the quality of the recording. GABA_A_R-mediated currents are blocked by PTX. **H:** Response failure rate at V_hold_ of + 40 mV and -70 mV (V_hold_ = -70 mV: median = 0.73, IQR = 0.24. V_hold_ =  + 40 mV, median = 0.53, IQR = 0.4. Age: P7-P10. n = 19 cells. *P* = 0.01 (*), Wilcoxon signed rank test.) (**A–E**) data are from electroporated WT animals that were subject to the same electroporation procedures.

Immature neurons in multiple brain regions have excitatory synapses with only NMDARs^23–25,41^. We thus verified whether L5 excitatory neurons in the TeA also have NMDAR-only connections at young ages. We identified NMDAR-only connections using LSPS^25,41^. AMPAR-mediated connections were recorded at a holding potential of -70 mV, while both AMPAR- and NMDAR-mediated connections were recorded at a holding potential of + 40 mV (Fig. 2A). Connections were classified as NMDAR-only if EPSCs were only detected at a holding potential of + 40 mV (Fig. 2B). We then computed the average input map for NMDAR-only connections for the population of cells at P6-7. This average input map showed that L5 neurons at P6-7 received NMDAR-only connections from stimulus locations that were located further away in the translaminar and intralaminar directions (Fig. 2C). Separating the laminar location of the connections revealed that lower layers tended to provide more NMDAR-only connections compared to AMPAR-mediated connections (Fig. 2D). Connections coming from lower layers had more NMDAR-only connections as quantified by the NMDAR-only to AMPAR-mediated connection index (Fig. 2E). By the end of the second postnatal week, more AMPAR-positive connections emerged from lower layers (Fig. 1E and F).

LSPS can only detect NMDAR-only connections that are outside of the regions with AMPAR-mediated connections, because we have to exclude regions with AMPAR-mediated connections. As an alternative, we used a minimal stimulation protocol to confirm the existence of NMDAR-only synapses in young animals (P6-P10). We performed minimal electrical stimulation of local axon terminals while recording EPSCs in young L5 neurons (Fig. 2F). We adjusted the stimulation strength such that around 70% of stimulation trials fails to evoke EPSCs in the recorded neurons^19^. Holding the neuron at + 40 mV revealed more synaptic connections, as evidenced by increased EPSC events and a decrease in the response failure rate (Fig. 2H). The increase in EPSCs recorded at + 40 mV was blocked by D-APV, an NMDAR blocker (Fig. 2G), suggesting that the newly revealed connections at + 40 mV holding potential were mediated by NMDAR-only synapses. Together, the results demonstrate that young L5 excitatory neurons have extensive NMDAR-only connections.

### Absence of NMDAR-mediated currents in *Grin1* knockout neurons

**Figure 3:**
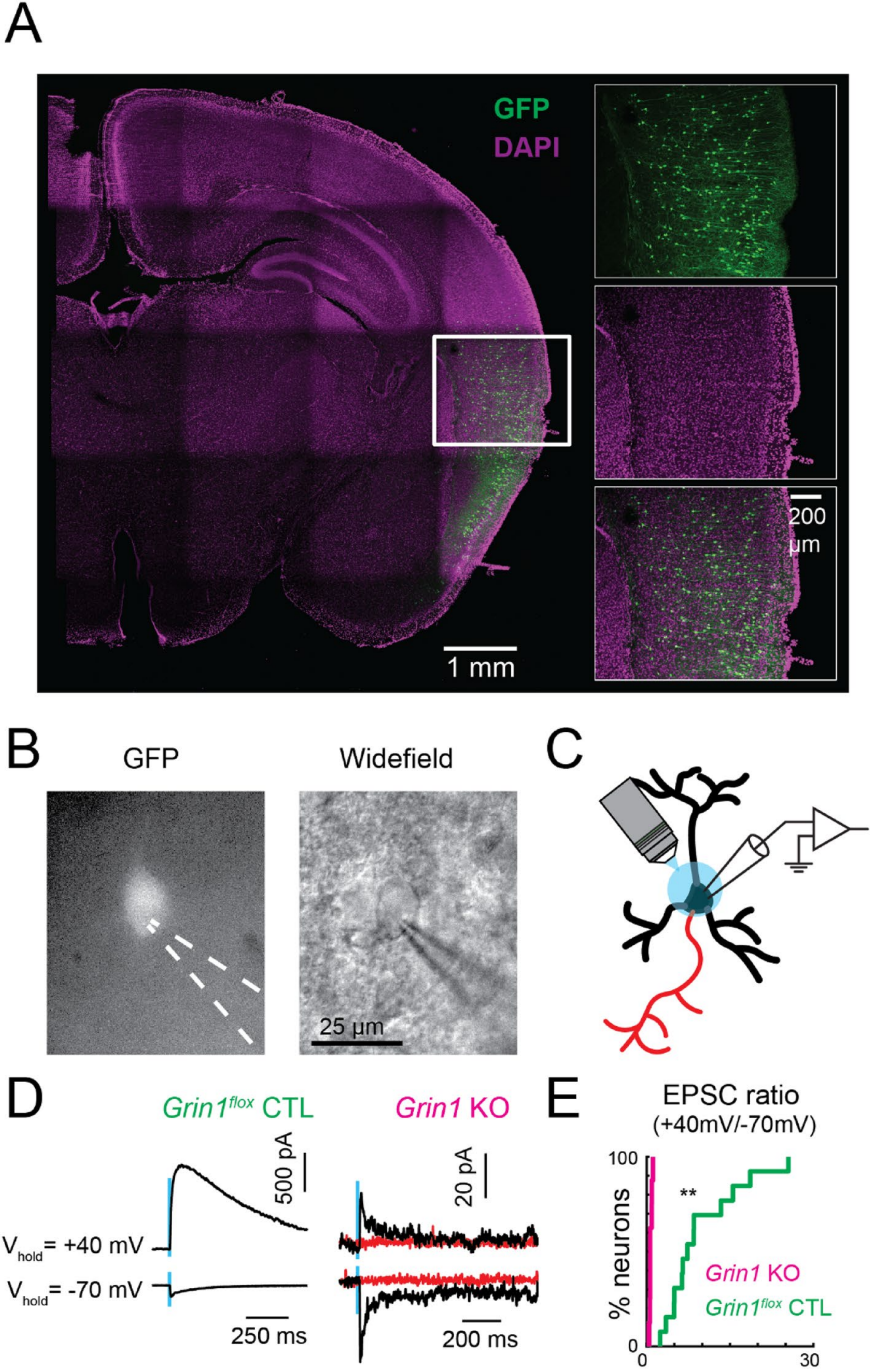
Absence of NMDAR-mediated currents in *Grin1* KO neurons. (**A**) Representative images of electroporated neurons in the TeA of *Grin1*^*flox*^ animals at P15. Green, immunofluorescence staining against GFP; magenta, DAPI stained cell nuclei. Magnified images of the boxed area show the laminar distribution of the electroporated neurons in the TeA. (**B**) Patch-clamp recording of an electroporated neuron identified by GFP expression. Left: epifluorescence image; right: transmitted light image. (**C**) Illustration of directly stimulating glutamate receptors on the recorded neuron through LSPS glutamate uncaging near the soma of the recorded neuron at young age (P6-7). AMPAR- and NMDAR-mediated EPSCs are recorded from whole-cell recordings. GABA_A_R-mediated currents are blocked by PTX; action potentials are blocked by TTX. (**D**) Directly activated EPSCs recorded from control (*Grin1*^*flox*^ control) and electroporated (*Grin1* KO) neurons from P6-7 animals. All currents from GFP + neurons can be blocked by NBQX (red traces). Blue vertical line marks the time of stimulation (1 ms duration). (**E**) Cumulative distribution plot of the ratio of the peak currents recorded at + 40 mV and -70 mV holding potential in control (*Grin1*^*flox*^ control) and electroporated (*Grin1* KO) neurons. *Grin1* KO: median = 0.66, IQR = 0.28, n = 13 cells. *Grin1*^*flox*^ control: median = 7.36, IQR = 8.9, n = 8 cells. *P* < 0.001 (**), Wilcoxon rank sum test.

To investigate the role of NMDARs in the development of synaptic connections, we generated mosaic *Grin1* (encoding the obligatory GluN1 subunit) knockout of excitatory neurons through in utero electroporation of *Cdk5r*-Cre-IRES-GFP plasmid in *Grin1*^*flox*^ pups at embryonic day (E)12.5 or E13.5. The *Cdk5r* promoter was used to restrict the deletion to postmitotic pyramidal neurons^50^. After electroporation, the targeted region (i.e., TeA) contained a small population of transfected neurons (GFP-positive, GFP + , *Grin1* knockout) and abundant non-transfected neurons (GFP-negative, GFP-, *Grin1*^*flox*^ control) (Fig. 3A). To test if GFP + neurons lacked NMDAR-mediated currents, we recorded EPSCs in response to focal glutamate uncaging onto both kinds of neurons at holding potentials of −70 mV and + 40 mV to delineate both AMPAR- and NMDAR-mediated currents (Fig. 3B and C). At P6-7, the GFP + , putative *Grin1* knockout (KO) neurons had intact AMPAR-mediated responses (peak amplitude in *Grin1*^*flox*^ control: median 187 pA, IQR 152 pA, n = 13 cells; *Grin1* KO: median 163 pA, IQR 240 pA, n = 8 cells. P = 1, Wilcoxon rank-sum test). As expected, GFP + neurons had greatly reduced responses at holding potential of + 40 mV compared to GFP- neurons in the same slice (Fig. 3D). In particular, long-lasting currents were abolished and remaining currents were similar to currents observed at −70 mV (Fig. 3D right). These results indicate a lack of NMDARs in GFP + neurons. We quantified the effect of electroporation by calculating the ratio of peak amplitudes between currents recorded at + 40 mV and −70 mV holding potentials. The peak current ratio is greatly reduced in GFP-positive neurons (Fig. 3E). Moreover, in GFP + neurons, the remaining current at + 40 mV holding potential was completely blocked by NBQX, the AMPAR-specific blocker (Fig. 3D right), indicating that the remaining current at + 40 mV was carried through AMPARs. Together, these data confirmed that our electroporation approach completely abolished NMDAR-mediated currents in GFP + neurons.

### Development of AMPAR-mediated connections is largely unchanged in the absence of NMDARs

**Figure 4:**
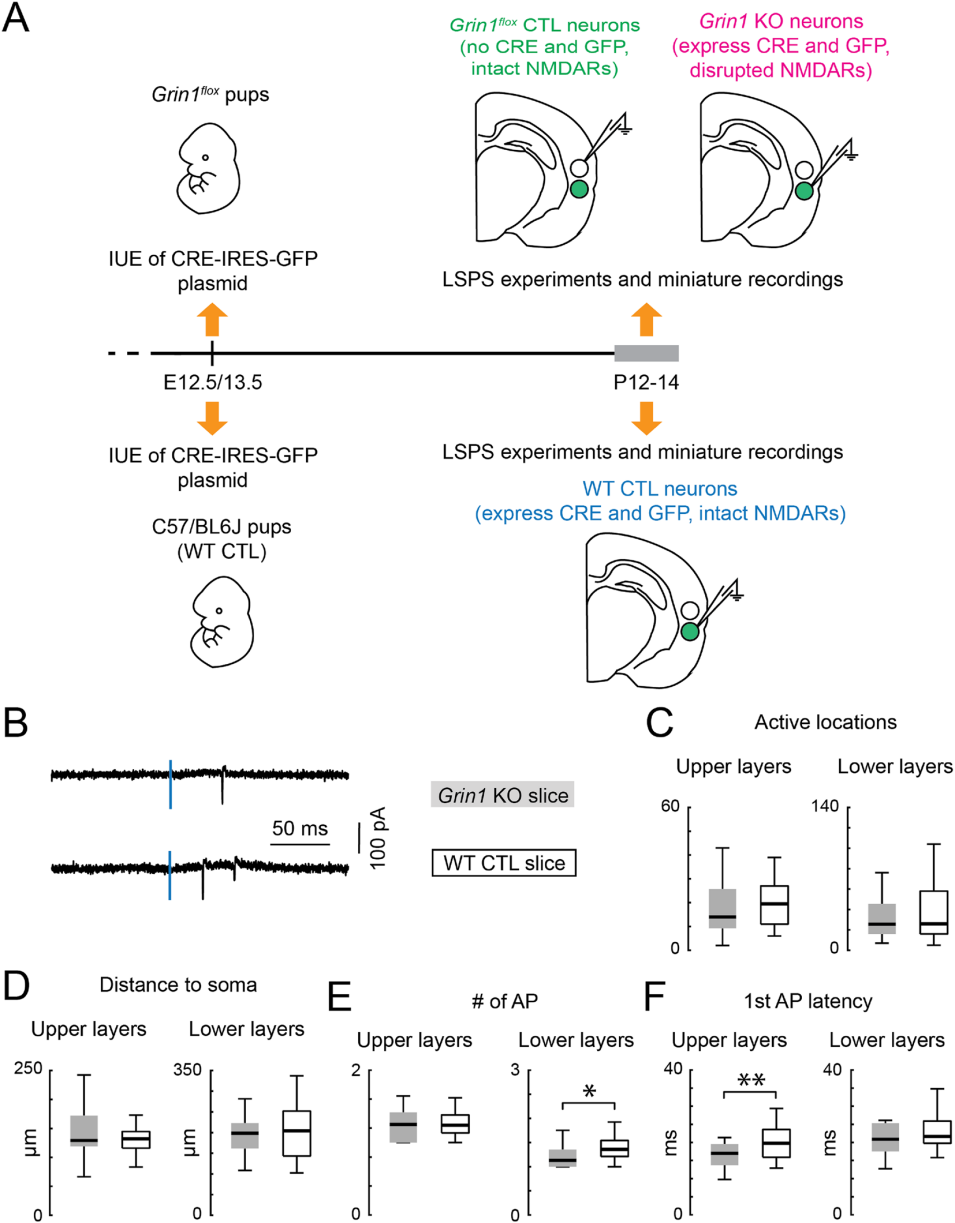
Similar spatial resolution of glutamate uncaging between *Grin1* KO and WT control slices. (**A**) Cartoon illustration of the major experimental procedures. *Grin1*^*flox*^ and C57BL/6 J (WT CTL) pups were electroporated on embryonic day 12.5 or 13.5. Pup brains with successful electroporation were sliced on postnatal day (P) 12–14 for patch-clamp recordings. *Grin1* KO neurons were GFP positive neurons from electroporated *Grin1*^*flox*^ pups. They express CRE and GFP proteins, and lack NMDAR-mediated currents (tested on P6-7). *Grin1*^*flox*^ neurons were GFP negative neurons adjacent to *Grin1* KO neurons. They don’t express CRE and GFP, and have intact NMDAR-mediated currents. WT CTL neurons were GFP positive neurons from electroporated C57BL/6J pups. They express CRE and GFP, and have intact NMDAR-mediated currents. All recorded neurons were located in the middle layer of the TeA region. The size of the neurons in the cartoon was magnified for illustration purpose. Loosely-attached recordings were also performed on P12-14. (**B**) LSPS-evoked action potentials revealed by cell-attached recording of neurons in the TeA. The vertical blue line indicates the uncaging time point. (**C**) Box plot of the number of locations where action potentials (AP) can be evoked by glutamate photostimulation of the cortical excitatory neurons. (**D**) Box plot of the distance within which 80% of action potentials were evoked in cortical excitatory neurons. (**E**) Box plot of the average number of action potentials evoked by glutamate photostimulation of the cortical excitatory neurons. Lower layers: *Grin1* KO slice vs WT control slice, *P* = 0.02. (**F**) Box plot of the average latency to first action potential evoked by glutamate photostimulation of the cortical excitatory neurons. Neurons are separated into upper layer group and lower layer group based on their soma location relative to the middle of the cortex. Upper layers: *Grin1* KO slice vs WT control slice, *P* = 0.006. *Grin1* KO slice: upper layers, n = 23 cells; lower layers, n = 20 cells. WT control slice: upper layers, n = 30 cells; lower layers, n = 26 cells. *P* < 0.01 (**), *P* < 0.05 (*), otherwise *P* > 0.05. Wilcoxon rank sum test. Brain slices are from P12-14 *Grin1*^*flox*^ and WT animals after in utero electroporation.

We next investigated if the development of translaminar connections mediated by AMPARs (Fig. 1E) requires NMDARs. We electroporated *Grin1*^*flox*^ pups at E12.5 or E13.5 and investigated the locations of AMPAR-mediated connections to L5 neurons in the TeA at P12-P14 through whole-cell voltage clamp recording and LSPS (Fig. 4A). Since we generated mosaic *Grin1* KO slices through in utero electroporation, we recorded GFP + *Grin1* KO neurons (*Grin1* KO), as well as GFP- *Grin1*^*flox*^ control neurons (*Grin1*^*flox*^ control) in the vicinity of the *Grin1* KO neurons. As over-expressing Cre and GFP might alter neuronal development, we performed an additional control by also electroporating C57BL/6 J wildtype pups with the same plasmid at E12.5 or E13.5 and then recording from GFP + neurons (WT control) (Fig. 4A). Thus, all three groups of cells were from pups that underwent the same surgical manipulations. *Grin1* KO and WT control both expressed Cre and GFP (thus controlling for Cre and GFP expression). Both *Grin1*^*flox*^ control and WT control neurons had intact NMDAR currents, and were compared to *Grin1* KO neurons to test the requirement of NMDARs during cortical development.

Proper interpretation of LSPS mapping experiments requires that the presynaptic response to glutamate uncaging remains similar between experimental conditions. Changes in a neuron’s response to glutamate uncaging may change the spatial resolution of the stimulus and confound the interpretation of the results. To rule out this possibility and to ensure that neurons were activated equally, we performed cell-attached recordings (Fig. 4B) from both transfected and non-transfected neurons across all layers in transfected slices from *Grin1*^*flox*^ and WT animals while performing glutamate uncaging. We divided neurons into two groups (i.e., upper and lower layers) based on their soma locations in relation to the middle of the cortex. To measure the spatial resolution of our stimulus, we quantified the total number of stimulus locations where action potentials were evoked by our stimulus (Fig. 4C) and the distance from the soma that encompasses 80% of those locations (Fig. 4D). We found no significant difference between *Grin1* KO slices and WT control slices, suggesting that the spatial resolution of our stimulus did not change. Small changes were detected in the average number of evoked action potentials from neurons in the lower layers (Fig. 4E) and in the average first spike latency from neurons in the upper layers (Fig. 4F). The change in the number of evoked action potentials was < 0.5 spike and thus did not influence measures of evoked PSC strength. The increases in latency were small (< 5 ms) and APs still fell within our time window of counting synaptic EPSCs. Thus, these changes did not interfere with the spatial resolution of our LSPS technique.

**Figure 5:**
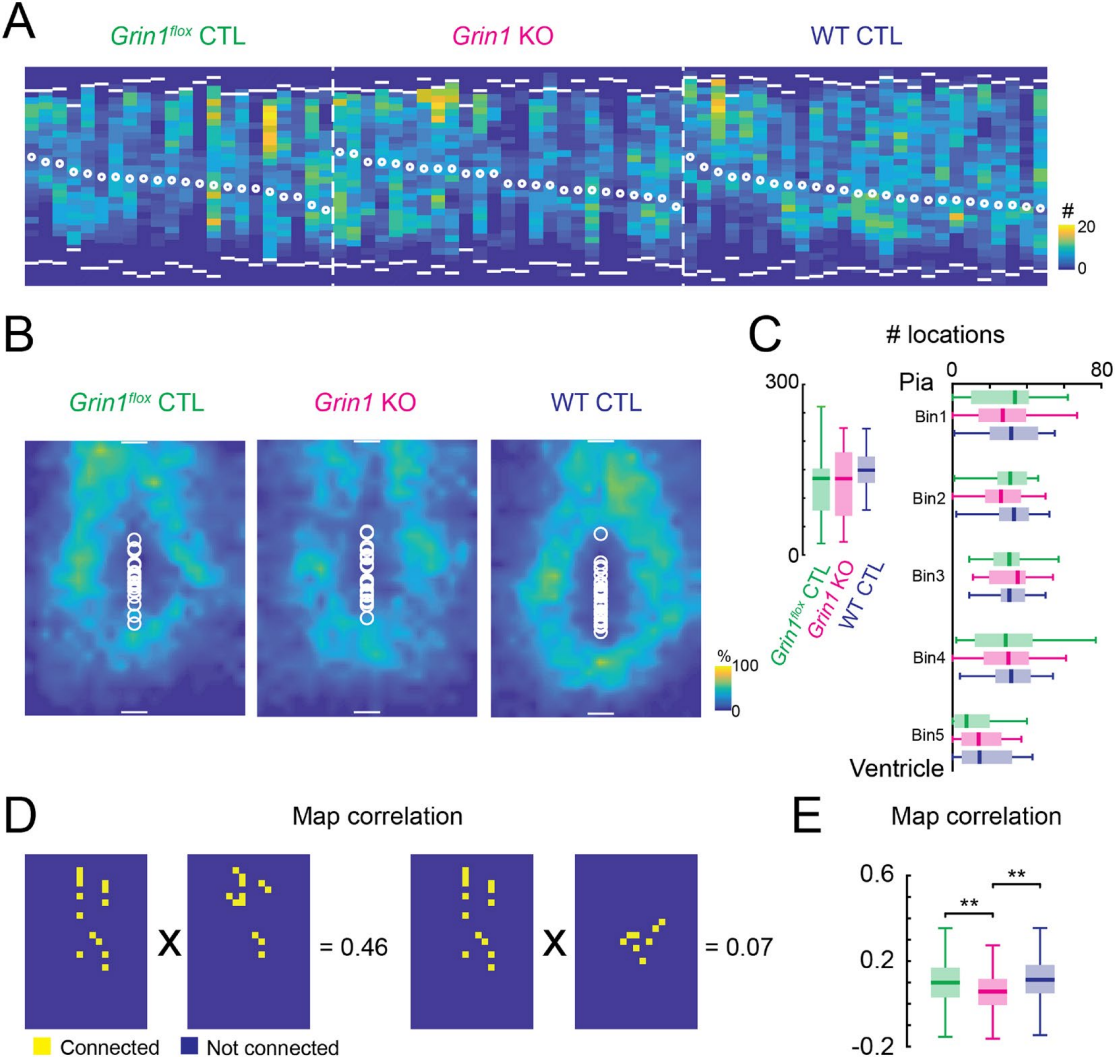
Development of AMPAR-mediated connections onto L5 excitatory neurons is largely unaffected in the absence of NMDARs. (**A**) Laminar distribution of AMPAR-mediated connections (evoked responses from whole-cell recordings) for each recorded neuron. Each column represents data from one neuron. White bars mark the pia (top) and ventricle (bottom) positions. White circles mark soma locations. Color indicates the number of effective stimulus locations. *Grin1*^*flox*^ control, n = 22 cells; *Grin1* KO, n = 25 cells; WT control, n = 26 cells. (**B**) Average AMPAR-mediated connection maps. White bars mark the pia (top) and ventricle (bottom) positions and represent 100 µm. White circles mark soma locations. Color indicates the fraction of cells that have synaptic inputs from the different cortical locations. (**C**) Box plots of the total number of effective stimulus locations for AMPAR-mediated connections. Left: total; right: laminar distributions. All *P* > 0.05. (**D**) Schematic illustration of how pairwise correlation between individual connection maps is calculated. Yellow squares indicate connected locations on the individual connection map; blue squares indicate locations with no connections. (**E**) Pairwise correlations of AMPAR-mediated connection maps for *Grin1*^*flox*^ control, *Grin1* KO and WT control neurons. *Grin1*^*flox*^ control vs *Grin1* KO, *P* = 4.6 × 10^–6^. WT control vs *Grin1* KO, *P* = 2.4 × 10^–9^. *P* < 0.01 (**), otherwise *P* > 0.05. Kruskal–Wallis test followed by Tukey's honest significant difference criterion for multi-group comparison was used in (**C**). One-way analysis of variance test followed by Tukey's honest significant difference criterion for multi-group comparison was used in **E**. For all box plots: Green, *Grin1*^*flox*^ control, n = 22 cells; red, *Grin1* KO, n = 25 cells; blue, WT control, n = 26 cells. Brain slices are from P12-14 *Grin1*^*flox*^ and WT animals after in utero electroporation.

We first investigated the laminar distribution of AMPAR-mediated connections for each cell and whether the removal of functional NMDARs would alter the distribution. To generate a laminar input profile for each cell we summed the AMPAR-mediated connections along the dorsal to lateral axis. We then generated a laminar input distribution plot for cells in each group (Fig. 5A). The laminar distribution of inputs revealed that individual cells in each group had diverse laminar distribution of the AMPAR-mediated inputs. AMPAR-mediated connections in all cell groups originate from both upper layers and lower layers of the cortex with no obvious differences among them. These results suggest that lack of functional NMDARs did not affect the gross laminar profile of AMPAR-mediated connections onto L5 excitatory neurons.

We next investigated if NMDAR removal altered the 2-dimensional pattern of connections. The average connection maps for cells in each group (Fig. 5B) were calculated as described in Fig. 1. Qualitative inspection of these average connection maps did not reveal gross differences between *Grin1* KO neuron and both groups of control neurons–suggesting that lack of functional NMDARs did not affect the gross 2-dimensional topology of AMPAR-mediated connections onto L5 excitatory neurons.

We next quantified these qualitative observations. To compare the amount of AMPAR-mediated input among these groups, we measured the total number of stimulus locations that provided input to cells in each group (Fig. 5C left). This analysis showed that *Grin1* KO and control cells received AMPAR-mediated connections from a similarly large area of cortex. We next quantified the laminar distribution of AMPAR-mediated connections. For each neuron we binned the cortical extent into 5 equal bins spanning from the pia to the bottom of L6. We then calculated the number of stimulus locations that provided input for each bin. This analysis revealed no significant difference in the laminar distribution of AMPAR-mediated connections among the three cell groups (Fig. 5C right), consistent with the qualitative observations. Together, these results suggest that the development of AMPAR-mediated inter-laminar connections does not require functional NMDARs.

Although the laminar analysis showed no significant differences, our laminar analysis emphasized comparison of the central tendency of the data but ignored the variability in the spatial connections at the individual cell level (Fig. 5A), which might be altered by knocking out NMDARs. To quantify the variability of the connection pattern between cells in the same group, we calculated the pairwise correlation of the AMPAR-mediated connection maps within each group (Fig. 5D). High correlation value indicates low variability in the connection patterns. There was a small but significant decrease of the correlation in *Grin1* KO neurons (Fig. 5E), suggesting that the connection patterns were more diverse after knocking out NMDARs. Further comparing the pairwise correlation of cells from the same slice (intra-slice) to that of cells from different slices (inter-slice), we did see consistent change of pairwise correlation for cells from the same slice (Fig. S1). Meanwhile, the three groups of data had similar average number of recorded cells per slice. Thus, the change in map correlations is not due to higher correlations for intra-slice comparison.

**Figure 6:**
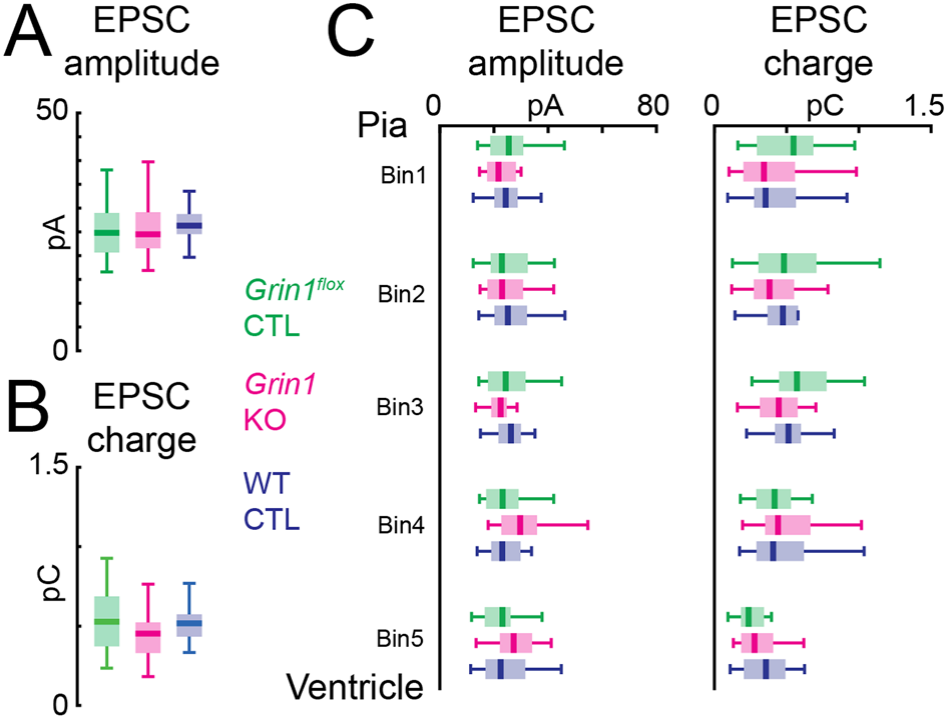
NMDAR knock-out does not change the strength of AMPAR-mediated connections onto L5 excitatory neurons. (**A**) Box plot of overall average EPSC peak amplitude. All *P* > 0.05. (**B**) Box plot of overall average EPSC charge. All *P* > 0.05. (**C**) Box plot of laminar distribution of average EPSC amplitude (left) and average EPSC charge (right). All *P* > 0.05. Kruskal–Wallis test followed by Tukey's honest significant difference criterion for multi-group comparison was used in (**A–C**). For all plots: Green, *Grin1*^*flox*^ control, n = 22 cells; red, *Grin1* KO, n = 25 cells; blue, WT control, n = 26 cells. Brain slices are from P12-14 *Grin1*^*flox*^ and WT animals after in utero electroporation.

Our analysis so far did not take into account the strength of the synaptic connections. To explore the possibility that NMDARs are involved in controlling the strength of AMPAR-mediated connections, we quantified the average peak amplitude and transferred charge of AMPAR-mediated EPSCs for each cell (Fig. 6A,B). This analysis revealed no significant changes among the three groups. Separately averaging peak amplitude and transferred charge for connections from different laminar locations (Fig. 6C) also showed similar distributions for all three groups. Thus, these analyses indicate that the strength of AMPAR-mediated connections is not changed in *Grin1* KO neurons. Together, the results suggest that the development of AMPAR-mediated connections onto L5 excitatory neurons in the TeA is largely intact in neurons without NMDARs, with a small increase in the variability of the connection patterns.

### Decreased GABA_A_R-mediated connections in the absence of NMDARs

**Figure 7:**
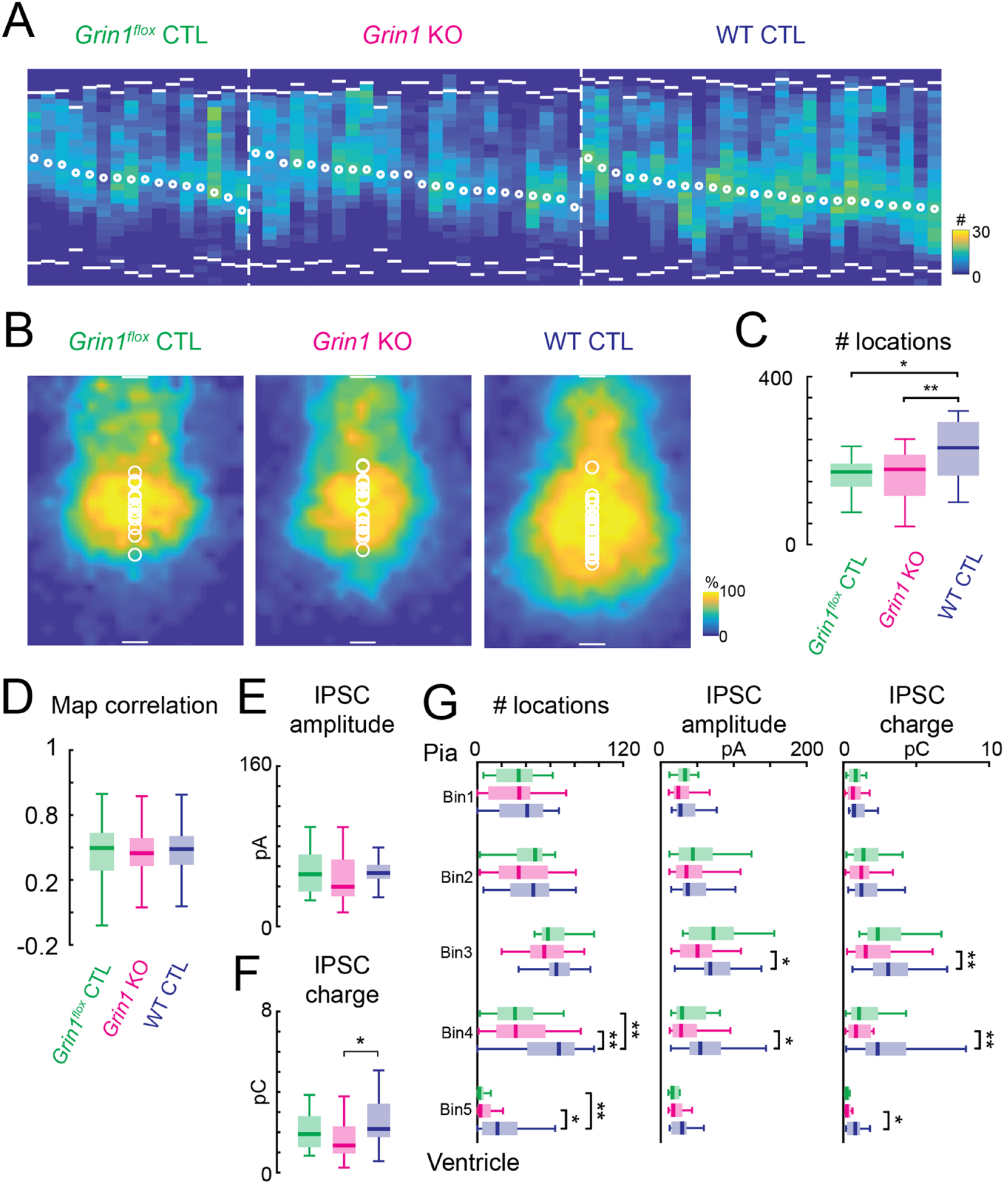
Decreased regional inhibition in the network after the loss of functional NMDARs. (**A**) Laminar distribution of GABA_A_R-mediated connections (evoked responses from whole-cell recordings) for each recorded neuron. Each column represents data from one neuron. White bars mark the pia (top) and ventricle (bottom) positions. White circles mark soma locations. Color indicates the number of effective stimulus locations. *Grin1*^*flox*^ control, n = 16 cells; *Grin1* KO, n = 24 cells; WT control, n = 26 cells. (**B**) Average GABA_A_R-mediated connection maps. White bars marks pia (top) and ventricle (bottom) positions and represent 100 µm. White circles mark soma locations. Color indicates the fraction of cells that receive inputs from the different cortical locations. (**C**) Box plot of total number of effective stimulus locations for GABA_A_R-mediated connections. *Grin1*^*flox*^ control vs WT control, *P* = 0.019. *Grin1* KO vs WT control, *P* = 0.009. (**D**)Pairwise correlations of GABA_A_R -mediated connection maps for *Grin1*^*flox*^ control, *Grin1* KO and WT control neurons. All *P* > 0.05. (**E**) Overall average IPSC peak amplitude. All *P* > 0.05. (**F**) Overall average IPSC transferred charge. *Grin1* KO vs WT control, *P* = 0.015. (**G**)Laminar distribution of the number of effective stimulus locations (left), average IPSC amplitude (middle), and average IPSC transferred charge for GABA_A_R-mediated connections. Left (number of effective stimulus locations): Bin4, *Grin1* KO vs WT control, *P* = 0.002; *Grin1*^*flox*^ control vs WT control, *P* = 0.002. Bin5, *Grin1* KO vs WT control, *P* = 0.022; *Grin1*^*flox*^ control vs WT control, *P* = 0.009. Middle (average IPSC amplitude): Bin3, *Grin1* KO vs WT control, *P* = 0.033; Bin4, *Grin1* KO vs WT control, *P* = 0.014. Right (average IPSC transferred charge): Bin3, *Grin1* KO vs WT control, *P* = 0.009; Bin4, *Grin1* KO vs WT control, *P* = 0.005; Bin5, *Grin1* KO vs WT control, *P* = 0.046. For C-G: *P* < 0.05 (*), *P* < 0.01 (**), otherwise *P* > 0.05. Kruskal–Wallis test followed by Tukey's honest significant difference criterion for multi-group comparison was used to test the significance of the difference in (**C**) and (**E–G**). One-way analysis of variance test followed by Tukey's honest significant difference criterion for multi-group comparison was used in (**D**). For all plots: Green, *Grin1*^*flox*^ control, n = 16 cells; red, *Grin1* KO, n = 24 cells; blue, WT control, n = 26 cells. Brain slices are from P12-14 *Grin1*^*flox*^ and WT animals after in utero electroporation.

Removal of NMDARs in hippocampal and cortical neurons can alter the development of GABA_A_R-mediated synaptic inputs^38–40^, and NMDARs can also be found at GABAergic synapses^51,52^. We therefore investigated whether the development of translaminar connections mediated by GABA_A_Rs requires NMDARs. We used LSPS glutamate uncaging to stimulate cortical neurons and isolated GABA_A_R-mediated IPSCs by holding L5 excitatory neurons at 0 mV (Fig. 1B). We first analyzed the laminar distribution of GABA_A_R-mediated connections for cells in each group by generating a laminar connection distribution plot (Fig. 7A). Similar to previous reports^48,49,53^, the laminar connections plot revealed that L5 pyramidal neurons mostly received GABA_A_R mediated connections from laminar locations close to the soma of the recorded L5 neurons, although connections can also come from distant locations. To reveal the 2-dimensional pattern of the locations of presynaptic cells, we generated the average input maps for GABA_A_R-mediated connections (Fig. 7B). The average input maps from *Grin1* KO and *Grin1*^*flox*^ control neurons were similar. However, both *Grin1* KO and *Grin1*^*flox*^ control groups appeared to have fewer connections when compared to the WT control group. Quantification of the total number of stimulus locations that gave rise to GABA_A_R-mediated connections (Fig. 7C) revealed that *Grin1* KO and *Grin1*^*flox*^ control neurons had fewer number of effective stimulus locations than WT control neurons, which confirmed our qualitative observations. Separately analyzing the number of GABA_A_R-mediated connections from different laminar locations revealed that the number of connected locations from the lower part of the cortex was significantly decreased for *Grin1* KO and *Grin1*^*flox*^ control neurons (Fig. 7G left). Together these results indicate that the lack of functional NMDARs in a subpopulation of excitatory neurons resulted in decreased regional GABA_A_R-mediated connections onto L5 excitatory neurons in the TeA.

To investigate if the variability of the GABA_A_R-mediated connections pattern changes after knocking out NMDARs, we computed the correlation of the connection maps. There was no significant difference among the three groups (Fig. 7D), suggesting that the diversity of the connection pattern did not change after losing NMDARs.

We also quantified the connection strength of GABA_A_R-mediated connections by calculating the average peak amplitude and transferred charge of GABA_A_R-mediated IPSCs for each cell. Overall, we found no differences in peak amplitude between groups (Fig. 7E), but *Grin1* KO neurons showed lower transferred charge compared to WT control neurons (Fig. 7F). Separately comparing peak amplitude and transferred charge of GABA_A_R-mediated connections from different laminar locations revealed that *Grin1* KO neurons had reduced peak amplitude and transferred charge for connections coming from the middle and lower part of the cortex (Fig. 7G middle and G right). Thus, this analysis suggests that the strength of inhibitory connections to *Grin1* KO neurons is decreased. Since our cell-attached recordings did not show any differences between groups, the decrease of GABA_A_R-mediated connections observed in *Grin1* KO and *Grin1*^*flox*^ control groups were due to the change in the connected locations. Together, our results suggest that the development of regional inhibition onto L5 excitatory neurons in the TeA requires intact NMDAR-mediated signaling.

### Altered excitation/inhibition balance after mosaic NMDARs knockout

**Figure 8:**
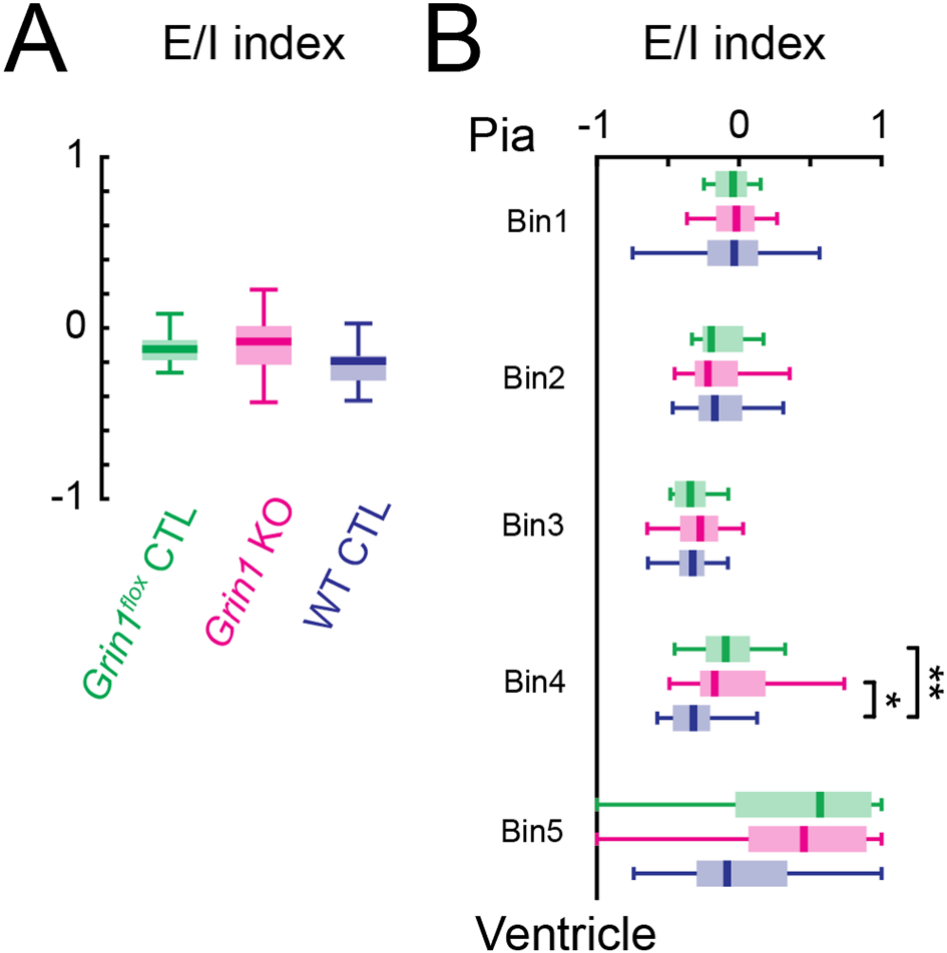
Altered regional excitation-inhibition index after the loss of functional NMDARs. (**A**) Excitation/inhibition (E/I) index calculated using the number of stimulus locations that gave rise to excitation and inhibition in each cell (see methods). All *P* > 0.05. (**B**) Laminar distribution of the excitation/inhibition index. Altered excitation/inhibition index after the loss of functional NMDARs. Bin4: *Grin1*^*flox*^ control vs WT control, *P* = 0.016. *Grin1* KO vs WT control, *P* = 0.009. For A and B: Green, *Grin1*^*flox*^ control, n = 16 cells; red, *Grin1* KO, n = 24 cells; blue, WT control, n = 26 cells. *P* < 0.01 (**), *P* < 0.05 (*), otherwise *P* > 0.05. Kruskal–Wallis test followed by Tukey's honest significant difference criterion for multi-group comparison was used to test the significance of the difference. Brain slices are from P12-14 *Grin1*^*flox*^ and WT animals after in utero electroporation.

In adults, neurons in the central nervous system normally receive balanced excitatory and inhibitory inputs. Although normal AMPAR-mediated connections were observed in *Grin1* KO brain slices, the decrease of GABA_A_R-mediated connections from lower layers indicate a shift in the balance between excitation and inhibition from specific laminar locations. To test our hypothesis, an excitation/inhibition balance index was calculated from the number of effective stimulus locations for AMPAR- and GABA_A_R-mediated connections in the recorded neurons (Fig. 8). The overall excitation and inhibition index between *Grin1* KO neuron and *Grin1*^*flox*^ control neurons was similar (Fig. 8A), but both *Grin1* KO neuron and *Grin1*^*flox*^ control neurons showed an increase in excitation/inhibition index from lower layers of the cortex compared to WT control neurons (Fig. 8B). These results suggest that our mosaic NMDAR deletion caused an imbalance of excitation and inhibition arising from lower cortical areas in the TeA.

**Figure 9:**
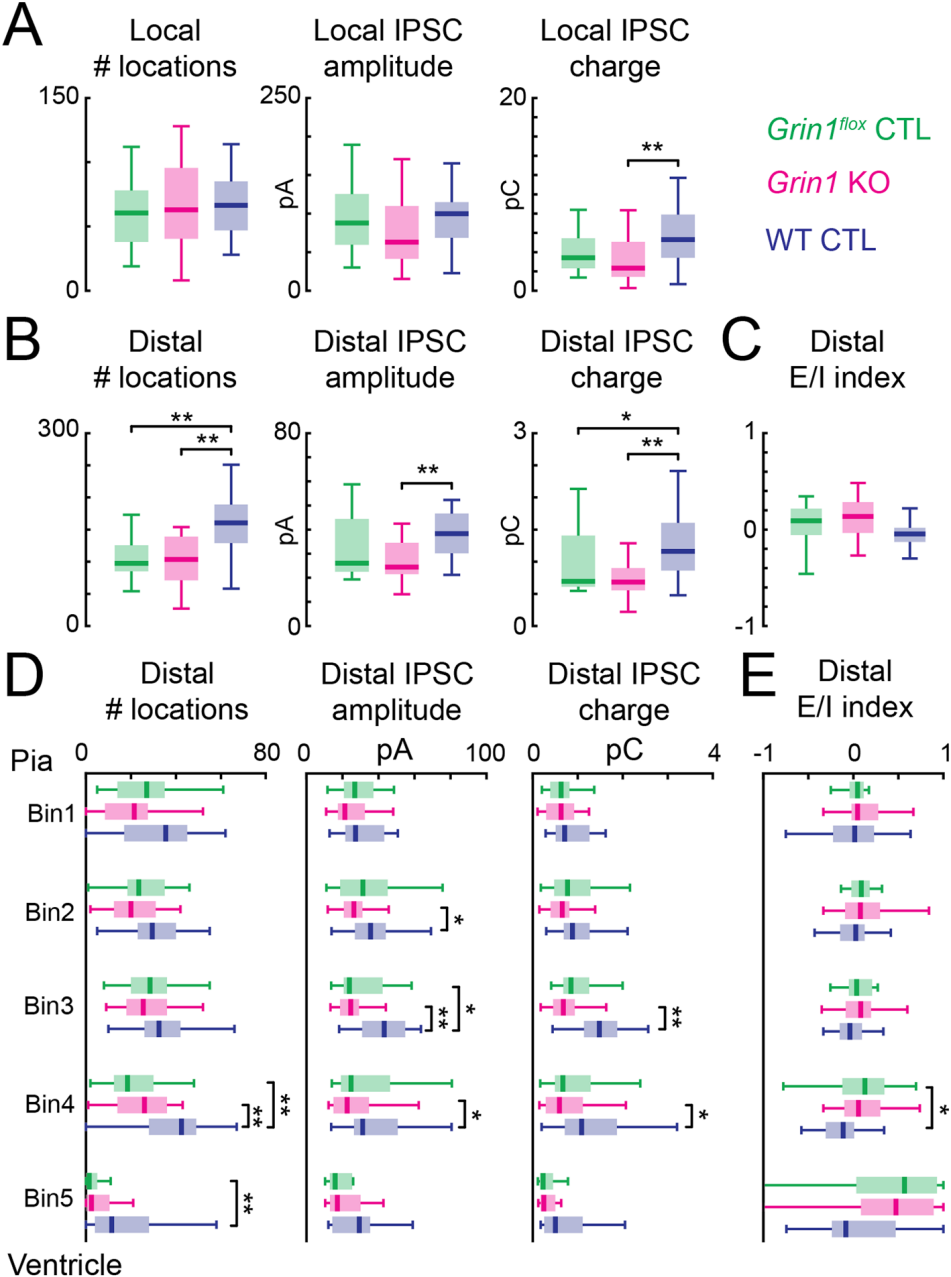
Distal GABA_A_R-mediated connections were preferentially affected after NMDAR knock-out. (**A**) Local GABA_A_R-mediated connections (which fall within the range of direct AMPAR responses) were comparable among *Grin1* KO and control groups. Box plot of the total number of local effective stimulus locations (left), average IPSC peak amplitude (middle), and average IPSC transferred charge (right) for local GAGA_A_R-mediated connections. Right: *Grin1* KO vs WT control, *P* = 0.005. All others, *P* > 0.05. (**B**) Distal GABA_A_R-mediated connections (which are outside the range of direct AMPAR response) were reduced in *Grin1* KO neurons. Total number of effective stimulus locations (left), average IPSC amplitude (middle), and average IPSC transferred charge (right) for distal GAGA_A_R-mediated connections. Left: *Grin1*^*flox*^ control vs WT control, *P* = 0.009; *Grin1* KO vs WT control, *P* = 0.0006. Middle: *Grin1* KO vs WT control, *P* = 0.003. Right: *Grin1*^*flox*^ control vs WT control, *P* = 0.044; *Grin1* KO vs WT control, *P* = 0.0007. (**C**) Excitation-inhibition (E/I) index calculated using the number of stimulus locations that gave rise to distal excitation and inhibition in each cell (see methods). All *P* > 0.05. (**D**) Laminar distribution of the number of effective stimulus locations (left), average ISPC peak amplitude (middle), and average IPSC transferred charge (right) for distal GABA_A_R-mediated connections. Left: Bin4, *Grin1*^*flox*^ control vs WT control, *P* = 0.002; *Grin1* KO vs WT control, *P* = 0.002. Bin5: *Grin1*^*flox*^ control vs WT control, *P* = 0.001. Middle: Bin2, *Grin1* KO vs WT control, *P* = 0.042. Bin3, *Grin1*^*flox*^ control vs WT control, *P* = 0.02; *Grin1* KO vs WT control, *P* = 0.0003. Bin4, *Grin1* KO vs WT control, *P* = 0.022. Right: Bin3, *Grin1* KO vs WT control, *P* = 0.0003. Bin4, *Grin1* KO vs WT control, *P* = 0.03. (**E**) Laminar distribution of the excitation/inhibition index calculated from distal GABA_A_R-mediated connections. Bin4: *Grin1* KO vs WT control, *P* = 0.036. For all plots: Green, *Grin1*^*flox*^ control, n = 16; red, *Grin1* KO, n = 24; blue, WT control, n = 26. *P* < 0.01 (**), *P* < 0.05 (*), otherwise *P* > 0.05. *Grin1*^*flox*^ control, n = 16 cells; *Grin1* KO, n = 24 cells; WT control, n = 26 cells. Kruskal–Wallis test followed by Tukey's honest significant difference criterion for multi-group comparison was used to test the significance of the difference. Brain slices are from P12-14 *Grin1*^*flox*^ and WT animals after in utero electroporation.

During LSPS glutamate uncaging, the glutamate receptors on the recorded neuron could be directly activated when the stimulation location was too close to the recorded neuron. Locations with AMPAR-mediated direct activations were not included in the analysis. Therefore, the most proximal AMPAR-mediated connections were masked by direct activations. Meanwhile, GABA_A_R-mediated connections were not affected by direct activation. This uneven sampling of the origins of AMPAR- and GABA_A_R- mediated connections might affect our results. To test this, we first separated GABA_A_R-mediated connections into local and distal connections based on the direct activation area of AMPAR-mediated connections. GABA_A_R-mediated connections within the direct activation area of AMPAR-mediated connections were labeled as local connections. For the local GABA_A_R-mediated connections, the number of effective stimulus locations and average IPSC peak amplitude were unaffected by knocking out NMDARs (Fig. 9A left and middle). There was a small decrease of average IPSC transferred charge when comparing *Grin1* KO and WT control neurons (Fig. 9A right). However, for distal connections, *Grin1* KO and *Grin1*^*flox*^ control neurons had fewer number of effective stimulus locations and less average IPSC transferred charge than WT control neurons (Fig. 9B left and 9B right). There was a small decrease of average IPSC peak amplitude (Fig. 9B middle). The data suggest that GABA_A_R-mediated connections from distal locations were preferentially affected by knocking out NMDA receptors. We further analyzed GABA_A_R-mediated distal connections from different laminar locations. Separately comparing the number of effective stimulus locations, IPSC peak amplitude, and IPSC transferred charge of GABA_A_R-mediated distal connections from different laminar locations revealed that *Grin1* KO and *Grin1*^*flox*^ control neurons had fewer number of effective stimulus locations, *Grin1* KO neurons had reduced peak amplitude and transferred charge in the middle and lower part of the cortex (Fig. 9D), which was similar to our results in Fig. 6. The data suggest that GABA_A_R-mediated connections from lower layers of the cortex were affected after losing NMDARs. Calculating excitation/inhibition index using GABA_A_R-mediated distal connections revealed altered excitation/inhibition balance for connections from lower layers of the cortex in *Grin1* KO neurons (Fig. 9C and E), suggesting a change in the excitation/inhibition balance from lower layers of the cortex. Thus, our observation of reduced GABA_A_R-mediated connections was not a result of including areas where the glutamate receptors on the recorded cell can be directly activated.

### mIPSCS but not mEPSCs are altered in the absence of NMDARs leading to altered excitation/inhibition balance of spontaneous synaptic transmission

The synaptic connections revealed by LSPS are action-potential-evoked synaptic transmissions^25,41^, and the glutamatergic connections from proximal neurons are masked by direct activation of the recorded cell by LSPS. Moreover, recent studies suggest that action-potential-evoked synaptic transmission and spontaneous synaptic transmission are generated through different mechanisms and can be regulated separately^54–56^. To investigate if our results held for all inputs and if spontaneous synaptic transmission to L5 excitatory neurons is affected by lack of NMDARs, we performed voltage clamp recordings to measure the miniature excitatory postsynaptic currents (mEPSCs) and miniature inhibitory postsynaptic currents (mIPSCs) from *Grin1* KO and control neurons.

**Figure 10:**
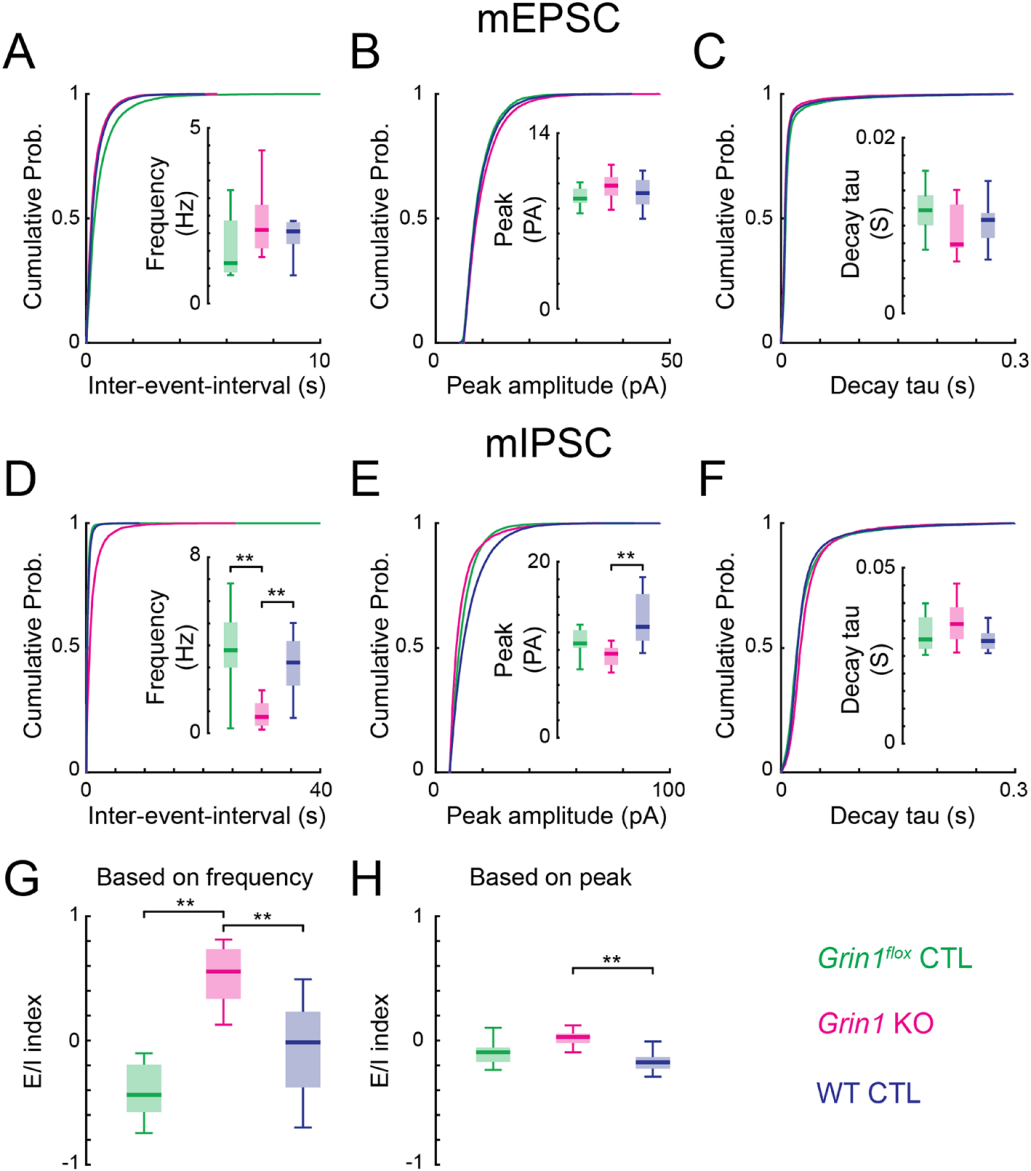
Spontaneous synaptic transmission in *Grin1* KO neurons. (**A**) Cumulative distribution of mEPSC inter-event-interval and box plot of average mEPSC frequency. (**B**) Cumulative distribution of mEPSC peak amplitude and box plot of average mEPSC peak amplitude. (**C**) Cumulative distribution of mEPSC decay time-constant and box plot of average mEPSC decay time-constant. (**D**) Cumulative distribution of mIPSC inter-event-interval and box plot of average mIPSC frequency. *Grin1*^*flox*^ control vs *Grin1* KO, *P* = 0.0006. WT control vs *Grin1* KO, *P* = 0.0007. (**E**) Cumulative distribution of mIPSC peak amplitude and box plot of average mIPSC peak amplitude. WT control vs *Grin1* KO, *P* = 0.0003. (**F**) Cumulative distribution of mIPSC decay time-constant and box plot of average mIPSC decay time-constant. For (**A–F**) *Grin1*^*flox*^ control, n = 11 cells; *Grin1* KO, n = 13 cells; WT control, n = 17 cells. (**G**) Boxplot of excitaton/inhibition (E/I) index calculated from the frequency of mEPSC and mIPSC. *Grin1*^*flox*^ control vs *Grin1* KO, *P* = 0.0001. WT control vs *Grin1* KO, *P* = 0.006. (**H**)E/I index calculated from the peak amplitude of mEPSC and mIPSC. WT control vs *Grin1* KO, *P* = 0.001. For G and H: *Grin1*^*flox*^ control, n = 11 cells; *Grin1* KO, n = 13 cells; WT control, n = 13 cells. *P* < 0.01 (**), *P* < 0.05 (*), otherwise *P* > 0.05. Cell average data were subject to Kruskal–Wallis test followed by Tukey's honest significant difference criterion for multi-group comparison. Brain slices are from P12-14 *Grin1*^*flox*^ and WT animals after in utero electroporation.

AMPAR-mediated mEPSCs were recorded at a holding potential of −70 mV. We quantified the frequency, peak amplitude and decay time constant of the recorded mEPSCs (Fig. 10A–C). *Grin1* KO neurons were similar to controls, suggesting that NMDARs are not required for the early development of spontaneous excitatory synaptic transmission to L5 excitatory neurons in TeA.

GABA_A_R-mediated mIPSCs were recorded at 0 mV holding potential. Similar to other studies^38–40^, we found that loss of NMDARs led to a significant decrease of mIPSC frequency (Fig. 10D). Electroporated WT neurons had significantly higher mIPSC peak amplitude than *Grin1* KO neurons (Fig. 10E). The decay time constant did not change between the *Grin1* KO and control neurons suggesting that the underlying subunit composition was similar (Fig. 10F). This suggests that the early development of spontaneous inhibitory synaptic transmission requires NMDARs.

The frequency and peak amplitude of mEPSCs and mIPSCs are related to the number and strength of the respective inputs. Thus, to quantify the excitation/inhibition balance of the spontaneous synaptic transmission, we used the frequency or the peak amplitude of mEPSC and mIPSC to calculate the excitation/inhibition index. The frequency-based excitation/inhibition index increased significantly in the *Grin1* KO neurons (Fig. 10G), but there was only a small increase in the peak amplitude-based excitation/inhibition index when comparing *Grin1* KO and WT control neurons (Fig. 10H). Together, our results suggest normal excitation/inhibition balance requires NMDARs.

## Discussion

We tested the requirement of NMDARs for the development of AMPAR- and GABA_A_R-mediated synaptic connections to L5 excitatory neurons in the temporal association cortex (TeA). Our results show that deletion of functional NMDARs from immature cortical excitatory neurons made the spatial pattern of AMPAR-mediated microcircuits more diverse without changing the amount of the connections. In contrast, NMDAR deletion in excitatory neurons caused a decrease in inhibitory connections. Action-potential-evoked and spontaneous inhibitory synaptic transmissions were differentially affected by NMDAR removal. For action-potential-evoked inhibitory synaptic transmission, both *Grin1* KO and *Grin1*^*flox*^ control neurons had a decrease in regional inhibitory connections, leading to altered regional excitation and inhibition balance. For spontaneous inhibitory synaptic transmission, only *Grin1* KO neurons had a decrease in inhibitory connections. Brain slices containing *Grin1*^*flox*^ control, *Grin1* KO, and WT control neurons were all subject to electroporation. Additionally, WT control neurons were transfected with the same Cre plasmid as *Grin1* KO neurons. Therefore, we can rule out that either electroporation or overexpression of Cre and GFP caused those changes. Thus, circuit maturation in the TeA can occur in the absence of NMDA receptors, but normal development of inhibition requires intact NMDAR signaling in these excitatory neurons.

Our results show that, as in the hippocampus, visual cortex, somatosensory cortex and auditory cortex, AMPAR-mediated connections onto L5 excitatory TeA neurons increase extensively during the first two postnatal weeks and that immature L5 excitatory neurons receive NMDAR-only connections^19–21,23–25,57–60^. Together, the evidence suggests that NMDAR-only connections and the development of AMPAR-mediated connections are general features of the early development of glutamatergic synaptic transmissions^31^.

Because activation of NMDARs in mature neurons can promote the insertion of AMPARs into the postsynaptic sites during long-term potentiation, it has been hypothesized that neuronal activity in immature neurons activates NMDAR-only synapses and facilitates the development of AMPAR-containing synapses^59,61–63^. Indeed, NMDAR-only connections are found to precede mature AMPAR-positive connections during development^23,25^, and NMDAR-only connections can be converted into AMPAR-positive connections^23,64^. However, several studies using genetic and pharmacological approaches to eliminate functional NMDARs have shown that functional AMPAR-containing synapses still develop in the absence of NMDARs^13,65–71^. Furthermore, activation of NMDARs could suppress EPSCs mediated by AMPARs in young neurons^66^, suggesting that NMDARs play a different role in regulating the developmental upregulation of AMPARs in immature neurons. Here, we investigated if NMDAR played a role in the early development of specific *circuits*. Our results show that neurons lacking NMDARs still have AMPAR-mediated EPSCs, consistent with prior observations^13,65,66,72^. We did not observe significant increases in the amplitude of AMPAR-mediated connections in *Grin1* KO neurons, possibly due to our probing of a variety of presynaptic sources or to the difference in the brain area under investigation^73^. Moreover, we found that removal of functional NMDARs did not alter the development and laminar topology of functional AMPAR-mediated circuits onto L5 excitatory neurons. However, our results show that the connection pattern is slightly more variable in *Grin1* KO neurons. Thus, together with prior studies, our results suggest that the development of AMPA-mediated circuits to L5 neurons is largely independent of postsynaptic NMDARs in these neurons.

We here use LSPS to probe neuronal connection. LSPS mapping methods cannot resolve the glutamatergic connections from nearby cells due to direct activation of dendrites of the recorded neurons. Thus, these local connections could be affected by the lack of NMDARs. However, our mEPSC recordings, which reflect all connections, were unaltered in excitatory neurons with NMDARs removed, suggesting that local connections are unaffected by lack of NMDARs. Additionally, we observed no difference in the decay time constant of the mEPSCs and mIPSCs. Since different subunit compositions underlie different kinetics of AMPA and GABA_A_ receptors^74–78^, disrupting NMDARs did not affect the subunit composition of AMPA and GABA_A_ receptors.

Similar to previous reports^38–40,79,80^, we observed a reduction of GABA_A_R-mediated synaptic transmission and a consequent change of E/I balance after disrupting NMDARs. As proper E/I balance is thought to maintain normal network activity and cortical functions^80–82^, it remains to be seen if the cortical network activity is changed in our *Grin1* knockout model. Furthermore, the effect of disrupting NMDARs on GABA_A_R-mediated synaptic transmission is not identical for action-potential-evoked and spontaneous synaptic transmissions. A decrease in action-potential-evoked GABA_A_R-mediated transmission was observed in both *Grin1* KO and *Grin1*^*flox*^ control neurons but a decrease in spontaneous GABA_A_R-mediated transmission was only found in *Grin1 KO* neurons. Our results confirmed several prior observations of impaired GABA_A_R-mediated activity when comparing *Grin1* KO to *Grin1*^*flox*^ control neurons in the same brain slice^38–40,79^, in particular decreased spontaneous mIPSC frequency. However, unlike the impairment of the spontaneous mIPSC, our LSPS experiments here show that action-potential-evoked GABA_A_R-mediated IPSCs are similar between *Grin1* KO and *Grin1*^*flox*^ control neurons. One possible explanation for this differential effect is that the spontaneous and evoked synaptic transmissions have different synaptic properties^54,55^. The synaptic sites giving rise to spontaneous mIPSCs and evoked IPSCs are partially segregated at the postsynaptic sites in the hippocampus^83^. Moreover, retinoic acid’s effect on the inhibitory synaptic transmissions in the somatosensory cortex is different for the spontaneous mIPSCs and evoked IPSCs^56^, indicating that different groups of synapses might underlie both events. Thus, our manipulation of NMDARs might only affect the part of inhibitory synaptic transmission that is segregated from the evoked synaptic transmissions. An alternative interpretation is that LSPS could fail to reveal changes in connections from very proximal neurons, because glutamate release on the soma of recorded neuron could activate large amount of glutamate receptors and affect the accuracy of the recording by having a large change in the input resistance^84^ and thus mask synaptic events. If this is the case, there could be an increase of proximal GABA_A_R-mediated connections in *Grin1*^*flox*^ control neurons so that when assessed by miniature recordings, the total number of GABA_A_R-mediated connections did not differ from the WT control neurons. For the action-potential-evoked inhibitory synaptic transmission, despite the reduced GABA_A_R-mediated connections from lower layers onto *Grin1* KO and *Grin1*^*flox*^ control neurons compared to WT control, GABA_A_R-mediated connections from different cortical laminae still develop. Thus, the effect of NMDAR signaling on the development of translaminar inhibitory circuits is mostly restricted to connections from lower layers, which might be mediated by specific classes of interneurons.

Both *Grin1*^*flox*^ and WT pups were subject to the same electroporation procedures. Moreover, the *Grin1*^*flox*^ and WT mice we used share similar C57BL/6 genetic background. Thus, the difference we observed between the electroporated *Grin1*^*flox*^ and WT pups were not due to the surgical procedures or the genetic background of the mice. How would deletion of NMDARs in a subset of excitatory neurons affects the development of inhibition in the network? A large fraction of activity in developing cortical networks is mediated by NMDARs^85,86^. Also, NMDAR removal reduces the correlation of spontaneous activity in L4 neurons of the somatosensory cortex^87^. Since neural activity is crucial for the normal development of inhibitory synaptic transmission^88–91^, we speculate that lack of NMDARs in a subset of excitatory neurons would alter network activity and the developmental trajectory of inhibitory connections. Alternatively, reduced network activity could lead to down regulation of inhibition through homeostatic plasticity^92,93^.

A caveat of all manipulations of gene expression using Cre is that the effect depends on Cre activity. We here prevent NMDA receptor expression by utilizing in utero electroporation of Cre. While we did not detect any NMDAR mediated currents at P6 it is possible that some NMDA signaling might have been present at earlier developmental, e.g. embryonic, ages and that potentially this very early expression drove AMPAR upregulation. However, since most AMPAR upregulation occurs after P6 our results suggest that such early potential influence might be minor.

Prior studies showed that deleting *Grin1* in all cortical excitatory neurons results in altered projections of whisker afferents and altered dendritic branching in mouse somatosensory thalamus and cortex^11–13^, suggesting a role for NMDARs in the shaping these anatomical circuits. However, it is unclear from these studies where in the circuit NMDARs are required. Our manipulation removed NMDARs in a relatively small subset of excitatory neurons. When the acutely prepared brain slices were inspected under the fluorescence microscope, roughly 25–50 electroporated cells (GFP +) could be found in one square millimeter area. However, due to the thickness of the slice, the observation in the acutely prepared brain slices could be an underestimate. In few electroporated animals when the brains were processed with immunostaining, the GFP + neurons could be as high as 290 cells per square millimeter (around 1/10 of the total cells in one square millimeter area, without compensating for tissue shrinkage during fixation). Therefore, NMDARs were present in the majority of excitatory and all inhibitory neurons, potentially on their axon terminals^51,52,94,95^. Given that we did not find significant changes in the amount of AMPAR-mediated connections between *Grin1* KO and *Grin1*^*flox*^ control neurons, we speculate that presynaptic NMDAR-mediated signaling might play a role in the refinement of neural circuits, or that deletion of NMDARs in all neurons disrupts global network activity patterns leading to morphological changes. One feature of developing neuronal circuits is the presence of spontaneous and emergent sensory activity evident as bursts, many of which are slow and which can synchronize local neuronal populations^96–105^. As the developing neurons are slightly depolarized, NMDARs with their relatively longer time constants might enhance participation of neurons in these activity bursts. In contrast, AMPAR-mediated connections integrate information on faster time scales. Thus, the presence of NMDAR-only synapses at early ages might be a developmental specialization to propagate slow activity patterns. It will be informative to monitor the cortical network activity after NMDAR deletion and test if the subsequent changes of synaptic connections are correlated with changes in the network activity. Since complete removal of NMDARs impairs development^11–13^, the effect of removing NMDARs on the development of neural connections might be correlated with the number of cells lacking NMDARs. Since the number of electroporated cells is variable in our experiments, we separated the data based on the number of GFP + neurons observed from the acutely prepared brain slices. We did not find any correlations between the amount of electroporated neurons and the AMPAR- and GABA_A_R-mediated connections from the LSPS experiments. Alternatively, the LSPS experiments might not be spatially sensitive enough to reveal potential subtle differences in circuit changes to the range of NMDAR deletion in our experiments.

To maintain consistency in the manipulated neurons, we performed electroporation in a small time-window (between E12.5 and E13.5). The electroporated neurons were distributed across different layers in the recorded region (TeA). This is likely because at this developmental stage the Tbr2 positive intermediate progenitor cells (IPCs) can give rise to both lower and upper cortical excitatory neurons^106–108^. We did not observe a gross difference in the locations of the electroporated neurons between the electroporated *Grin1*^*flox*^ and WT brains slices. We tried to target the neurons in the middle of the cortex (corresponding to the upper layer 5 neurons in the primary sensory areas) for our recordings, as it is more consistent to locate electroporated neurons in this area in our experiments. Combining the electroporation time-window and the morphology of the recorded neurons, our manipulation is likely restricted in the excitatory neurons. But given the diversity of the cortical excitatory neurons^109–111^, it is possible that the effect we observed is specific to the neurons we targeted. It will be informative to use other methods, such as layer-specific Cre lines or performing the *Grin1* deletion at different developmental stages, to manipulate different populations of the cortical excitatory neurons and test if the results are different.

In summary, our results show that in excitatory cortical L5 neurons postsynaptic NMDA receptors are not required for the early assembly and refinement of AMPA-mediated microcircuits, but are required for the development of inhibitory synaptic transmission.

## Materials and methods

All procedures were approved by the University of Maryland Institutional Animal Care and Use Committee to be in accordance with applicable guidelines and regulations. The study was carried out in compliance with the ARRIVE guidelines.

### Animals

*Grin1*^*flox*^ mice (strain #005246, Jackson Laboratories) and C57BL/6J (wild type or WT) mice (strain #000664, Jackson Laboratories) were used to generate timed-pregnant mice for in utero electroporation. Mouse pups of both sexes from postnatal day 6 (P6) to P14 were used for in vitro electrophysiology experiments, including 19 electroporated *Grin1*^*flox*^ mice, 11 electroporated wild type mice, and 8 wild type mice.

### In utero* electroporation*

Timed-pregnant mice were generated by pairing male and female mice for two days. Surgeries were performed on embryonic day 12.5 (E12.5) or E13.5 as described previously^112,113^. We used electroporation of C*dk5r*-Cre-IRES-GFP plasmid (courtesy from Dr. Paola Arlotta, Harvard University) to express Cre and GFP in postmitotic pyramidal neurons^50^. For each embryo, 0.3–0.45 µl plasmid DNA solution (DNA concentration 1 ~ 1.5 µg/µl) was injected into the left ventricle. Five square voltage pulses (35–37 V, 50 ms duration, 1 Hz) were delivered through a needle electrode (Nepa Gene, CUY610P4-1). GFP signals in cortical neurons could be detected as early as 3 days after electroporation (data not shown). At P12-14, GFP + neurons were scattered across cortical layers, likely because intermediate neuronal progenitors can give rise to both upper layer and lower layer cortical neurons^106,107^. The density of GFP + neurons in the recorded L5 area was around 25–50 cells/mm^2^, when acutely prepared slices were imaged under the epifluorescence microscope. As a control we electroporated C57BL/6 mice. We did not see a gross difference in the distribution pattern of GFP + neurons between sections from *Grin1*^*flox*^ and WT mice. A small percentage of brains displayed abnormally enlarged lateral ventricles which were not correlated with the genotype of the mouse or the side of electroporation; these brains were excluded from experiments.

### Immunohistochemistry and imaging

Electroporated animals at P14-15 were transcardially perfused with cold phosphate buffered saline (PBS) and 4% paraformaldehyde (Electron Microscopy Sciences) under deep anesthesia (Fluriso, VetOne). Perfused brains were fixed in 4% paraformaldehyde for 24 h before transferring to 30% phosphate-buffered sucrose solution for cryoprotection. Cryoprotected brains were sectioned with a freezing microtome (Leica) in the coronal plane at a thickness of 50 μm and stored in PBS until use. To identify GFP-immunopositive cells, immunohistochemistry was performed using standard protocols. Briefly, a blocking solution was prepared with 5% normal goat serum (Cat no. 5560-0007, SeraCare) and 0.3% Triton-X 100 (Sigma). Selected brain sections were incubated in the blocking solution at room temperature for 90 min before being incubated overnight in chicken-anti-GFP (1:1000, ab13970, Abcam) at 4 °C. The sections were washed three times before incubating in secondary antibodies (goat anti-chicken, labeled with Alexa Fluor 488, 1:500, A11039, Invitrogen). Sections were then mounted with antifade mounting medium containing DAPI (H-1200, Vectashield).

The sections were imaged with a confocal microscope (10 × lens, SP5 X, Leica); z-stacks were taken at 2048 × 2048 resolution in 3.0 μm steps, with the tile function. Images were then processed with Fiji software. Fluorescence intensities were modified for visualization purposes.

### Electrophysiology

Acute brain slices were made as described previously^41^. In brief, after euthanasia, mouse brains were sectioned with a vibratome (VT1200, Leica) in ice cold sucrose artificial cerebrospinal fluid (ACSF) containing (in mM): 212.7 sucrose, 2.6 KCl, 1.23 NaH_2_PO_4_, 26 NaHCO_3_, 10 glucose, 3 MgCl_2_, 1 CaCl_2_ (pH 7.35–7.4). 400 µm thick coronal slices were incubated at 30 ºC for 30 min then kept at room temperature in ACSF containing (in mM): 130 NaCl, 3 KCl, 1.25 NaHCO_3_, 10 glucose, 1.3 MgSO_4_ and 2.5 CaCl_2_ (pH 7.35–7.4). ACSF was equilibrated with 95% O_2_-5% CO_2_. During LSPS experiments, the recording chamber was superfused with high-divalent ACSF to reduce polysynaptic transmission. High-divalent ACSF contains (in mM): 124 NaCl, 5 KCl, 1.23 NaH_2_PO_4_, 26 NaHCO_3_, 10 glucose, 4 MgCl_2_ and 4 CaCl_2_. Electroporated cortical neurons expressing GFP were identified under the epifluorescence microscope. We recorded neurons in the middle layer (putative layer 5) of the temporal association area (TeA) because it is the most consistently electroporated area in our surgery. 1–2 adjacent slices containing the TeA were taken from each brain. Whole-cell voltage clamp recordings were performed at room temperature (21–24 °C) with 3–7 MΩ borosilicate recording electrodes filled with internal solution containing (in mM): 115 cesium methanesulfonate, 5 NaF, 10 EGTA, 9 CsCl, 3.5 MgATP, 0.3 NaGTP and 3 QX-314 (pH 7.25; 300 mOsm). Internal solution also contained 0.5% biocytin. We normally record from 2–3 cells per brain slice. Data were acquired with a voltage-clamp amplifier (Multiclamp 700B; Molecular Devices) and digitized using a DAQ board (NI PCI-6259, National Instruments) using EPHUS^114^ in MATLAB (Mathworks). Membrane potential was corrected for 10 mV of estimated liquid junction potential. Series resistance (R_s_) was typically 20–40 MΩ and was not compensated during recording. To identify ‘silent’ synapses, minimal electrical stimulations were delivered through an extracellular bipolar electrode at the rate of 0.1 Hz^19^. AMPAR-mediated currents were recorded at −70 mV. AMPAR- plus NMDAR-mediated currents were recorded at + 40 mV. GABA_A_R-mediated currents were blocked by picrotoxin (PTX, 100 µM) in the bath solution. 60 to 120 stimulation trials at each holding potential were used to calculate response failure rate. The spontaneous miniature EPSCs and IPSCs were recorded at the holding potential of -70 mV and 0 mV, respectively, in the presence of TTX (1.5 µM) at room temperature (21–24 °C). R_s_ was monitored during recording and cells were excluded from analysis when the change of R_s_ was greater than 20%. Loose seal cell attached (R_seal_ between 20 and 50 MΩ) recordings were used to record action potentials without washing out the intracellular content. Recording electrodes were filled with filtered ACSF during loosely cell-attached recordings.

### Laser-scanning photostimulation (LSPS)

LSPS was performed as described previously^25,41,49,115^. Caged glutamate [0.8 mM *N*-(6-nitro-7-coumarylmethyl)-L-glutamate]^116^ was added in the bath solution. Photostimulation (355 nm, 1-ms pulses; 3510–100, DPSS Lasers Inc.) was delivered through a 10 × water immersion objective (Olympus UMPLFLN10XW, numerical aperture 0.3). To map the locations of presynaptic cells, laser power on the specimen was ~ 24 mW. A rectangular array of up to 20 × 30 sites with 40 μm spacing covering the whole cortical length was stimulated once at 1 Hz in a pseudorandom order during whole-cell voltage clamp recording. This stimulation paradigm evokes action potentials in neurons at the stimulation sites with similar spatial resolution (~ 150 μm) during development^44^. For identifying NMDAR-only connections, AMPAR- and NMDAR- mediated currents were recorded at holding potentials of -70 mV and + 40 mV in the presence of picrotoxin. Putative monosynaptic excitatory postsynaptic currents (EPSCs) were differentiated from direct activation of the glutamate receptors on the recorded cell based on the EPSCs’ latency. EPSCs in the 10 ms to 50 ms poststimulation time-window were classified as putative monosynaptic EPSCs (Fig. 1B). A minimal peak amplitude of 10 pA was used to reduce false positive PSCs caused by spontaneous PSCs or the noise during recording. We used the last 100 ms of the 1 s recording trace to estimate the false positive rate of our PSC detection criteria. The false positive rates were low and were comparable among the three groups (AMPAR-mediated: *Grin1*^*flox*^ control, median 0.0025 Hz, IQR 0.007; *Grin1* KO, median 0.0055 Hz, IQR 0.0129; WT control, median 0.004 Hz, IQR 0.0086. GABA_A_R- mediated: *Grin1*^*flox*^ control, median 0.0089 Hz, IQR 0.019; *Grin1* KO, median 0.0029 Hz, IQR 0.007; WT control, median 0.0065 Hz, IQR 0.016. All *P* > 0.05. Kruskal–Wallis test was used and followed by Tukey's honest significant difference criterion for multi-group comparison). The classified direct activation and monosynaptic EPSCs have been verified by repeating the experiments in the presence of TTX in previous reports. Most of the classified direct responses (~ 85%) were TTX-resistant and most of the classified monosynaptic EPSCs (~ 90%) were blocked by TTX^25,41^. Because the responses were highly repeatable (> 90%)^25^, in most mapping experiments we only have one trial per recording condition per cell. For studying the AMPAR- and GABA_A_R- mediated connections, EPSCs and inhibitory postsynaptic currents (IPSCs) were measured at −70 mV and 0 mV, respectively. For measuring the + 40 mV/-70 mV current ratio of EPSC responses to glutamate uncaging (Fig. 3), we used low laser power (5 – 10 mW) glutamate uncaging around the soma of the recorded cell. In these experiments GABA_A_R-mediated currents were blocked by picrotoxin (100 µM) and action potentials in the brain slice were blocked by TTX (1 µM, Tocris). D-2-Amino-5-phosphonovalerate (D-APV, 50 µM, Tocris) was used to block NMDARs; 2,3-dioxo-6-nitro-1,2,3,4-tetrahydrobenzo[f]quinoxaline-7-sulfonamide (NBQX, 10 µM, Tocris) was used to block AMPARs. To verify that our stimulus resolution did not change between the electroporated *Grin1*^*flox*^ slices and the electroporated WT slices, loose-patch recordings were used to record action potentials from neurons across all layers of the cortex during glutamate uncaging. All drugs and chemicals were purchased from Sigma-Aldrich unless specified otherwise.

### Data analysis and statistics

Data were analyzed using custom scripts written in MATLAB as previously described^25,41,49^. Putative monosynaptic EPSCs and IPSCs were identified through the criteria above. AMPAR- and GABA_A_R- mediated connection maps were constructed based on the photostimulation locations and the recorded synaptic currents. NMDAR-only connection maps were constructed by subtracting connected locations in AMPAR-mediated connection maps from connection maps recorded at + 40 mV. To reveal the general patterns of the connected locations, maps from different cells were aligned to the pia and ventricle locations to calculate the fraction of cells that received a connection from each stimulation location. Subsequently, data from AMPAR- and GABAAR-connection maps were further used for calculating laminar distribution plots, map correlations, and E/I balance. To give an overview of the laminar distribution of AMPAR- and GABA_A_R-mediated inputs, we summed the number of input locations in individual input map along the direction parallel to the pia and plotted the laminar distribution of input locations for each cell. Traditional LSPS analysis divides the cortex into layers based on histological difference visible under IR illumination and analyzes inputs separately from each layer^25,41,42,44,49,117,118^. Because TeA does not show clear layer boundaries under IR illumination and it is disputable if TeA has layer 4, we devised an alternative way to quantify the laminar distribution of the connections. We evenly divided the cortex into 5 areas (bins) along the pia-to-ventricle axis and quantified the number of presynaptic locations, average peak amplitude, average transferred charge of the inputs, and excitation-to-inhibition index in each area. Each area covered roughly 200 µm distance along the pia to ventricle axis. Roughly speaking, the bins 1–2 correspond to the layer 1 to 4 and bins 3–5 correspond to the layer 5 and 6 in the primary sensory area. We also repeated all of our analyses when aligning the cells to their soma location and got similar results, indicating that our results were not affected by how we align the individual connection maps from different cells. To quantify the balance of excitation and inhibition from the glutamate uncaging data, we used the number of locations with AMPAR- (*nAMPAR*) and GABA_A_R-mediated (*nGABA*_*A*_*R*) connections to calculate the excitation-to-inhibition (E/I) index (*EII*) as follows:

$$EII = \frac{{nAMPAR - nGABA_{A} R}}{{nAMPAR + nGABA_{A} R}}$$

Similarly, excitation-to-inhibition balance of spontaneous miniature recordings was calculated, where the frequency or the peak amplitude of miniature excitatory postsynaptic currents (mEPSCs) and miniature inhibitory postsynaptic currents (mIPSCs) were used to quantify excitation and inhibition, respectively. To quantify the laminar distribution of NMDAR-only/AMPAR-mediated connections ratio (N/A index) at P6-7, we used similar approach to calculate NMDAR-only/AMPAR-mediated connections index.

Miniature EPSC and IPSC events were identified in a semiautomatic fashion. At least 4 min of stable recordings at the same holding potential were used for each cell. Locations of the PSC peaks were identified from low-pass filtered data. PSC events were extracted and compared to the scaled average PSC. PSC events with abnormally different rise and decay kinetics were excluded. Detected PSC events were plotted on the original recording traces and manually inspected.

Median and interquartile range (IQR) were reported as the descriptive analysis. For two-group comparison, a two-sample *t*-test or a Wilcoxon rank-sum test was used to test the significance of the difference. For three-group comparison, Kruskal–Wallis test or one-way analysis of variance was used, then followed by Tukey's honest significant difference criterion for multi-group comparison. For paired samples, Wilcoxon signed rank test was used. Significance level was marked as *P* < 0.05 (*) or *P* < 0.01 (**); otherwise *P* > 0.05. Test methods, significant *P* values and the number of cells are detailed in the figure legends. All tests were performed in MATLAB (Mathworks). Effect size *r*^119^ is reported for all pairwise statistical tests (Table S1-7). r > 0.5 indicates a large effect; 0.3 < r < 0.5 indicates a medium effect; r < 0.3 indicates a small effect.

## Supplementary Information

Supplementary Information.
