# Supplementary material for: Cortical inhibitory but not excitatory synaptic transmission and circuit refinement are altered after the deletion of NMDA receptors during early development
Source: Sci Rep. 2023 Jan 12;13:656. doi: 10.1038/s41598-023-27536-0 (PMC9837136; doi:10.1038/s41598-023-27536-0)
Supplement: Supplementary file 1 — Supplementary Information. [file 41598_2023_27536_MOESM1_ESM.pdf]

Cortical inhibitory but not excitatory synaptic transmission and circuit refinement are altered after the deletion of NMDA receptors during early development

Rongkang Deng [2, 3], Minzi Chang[1], Joseph P. Y. Kao[4], Patrick O. Kanold[1, 2]

1 Supplemental figure

7 Supplemental tables

Supplemental figure 1

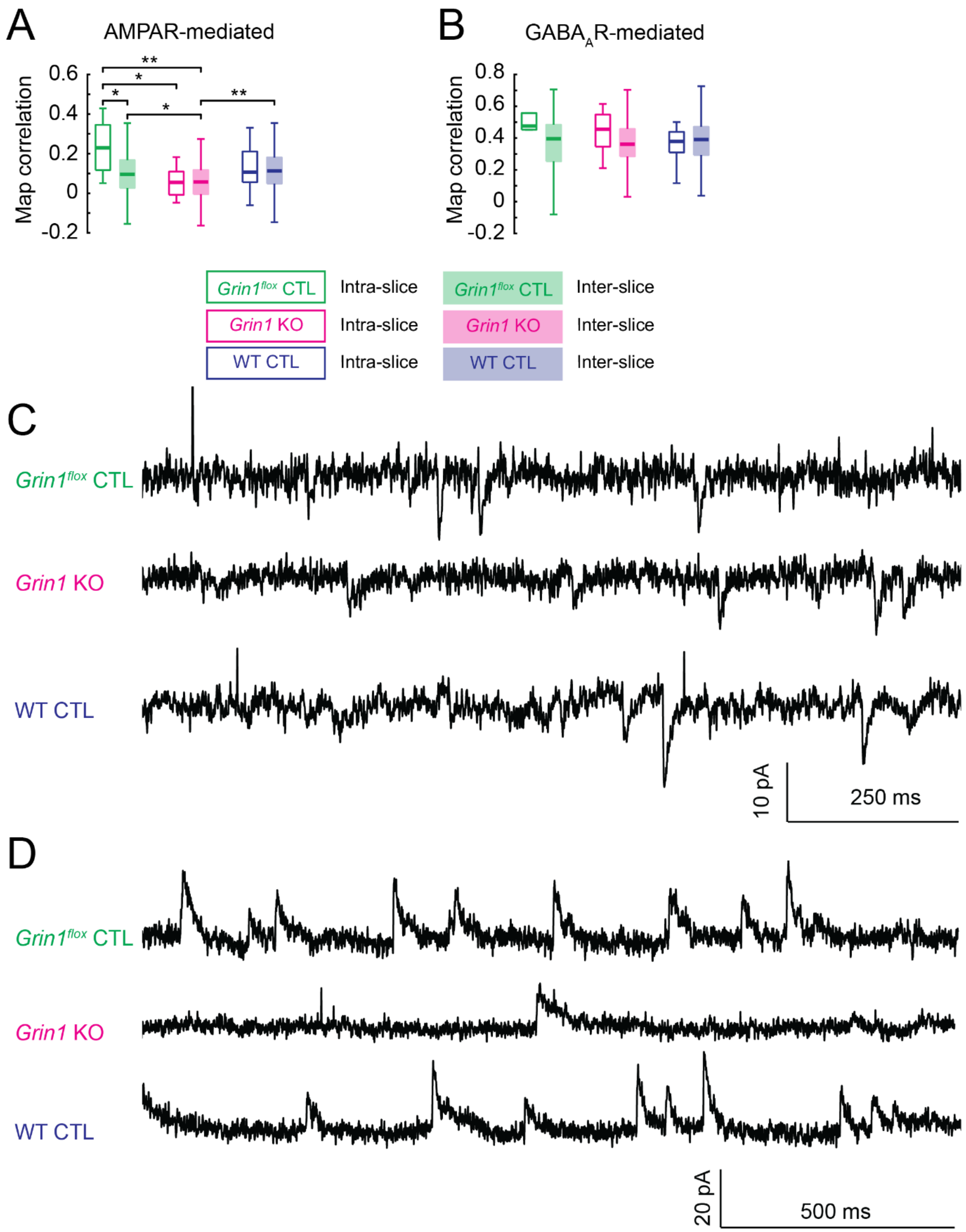

Figure S1. Intra- and inter-slice correlation of the connection maps.

A: Intra- and inter-slice correlation of AMPAR-mediated connection maps. *Grin1*^{flox} CTL intra- vs inter-, P = 0.027. *Grin1*^{flox} CTL intra- vs *Grin1* KO intra-, P = 0.027. *Grin1*^{flox} CTL intra- vs *Grin1* KO inter-, P = 0.0005. *Grin1*^{flox} CTL inter- vs *Grin1* KO inter-, P = 0.0015. *Grin1* KO inter- vs WT CTL inter-, $P = 2.3\times10^{-8}$. Intra-slice: *Grin1*^{flox} CTL, 2.25 cells (average) per slice in 8 slices; *Grin1* KO, 2.25 cells (average) per slice in 8 slices; WT CTL, 2.44 cells (average) per slice in 9 slices. Cell and slice numbers, Inter-slice: *Grin1*^{flox} CTL, 22 cells in 12 slices; *Grin1* KO, 25 cells in 15 slices; WT CTL, 26 cells in 13 slices. **B:** Intra- and inter-slice correlation of $GABA_A$R-mediated connection maps. All P > 0.05. Cell and slice numbers, Intra-slice: *Grin1*^{flox} CTL, 2.2 cells (average) per slice in 5 slices; *Grin1* KO, 2.13 cells (average) per slice in 8 slices; WT CTL, 2.44 cells (average) per slice in 9 slices. Inter-slice: *Grin1*^{flox} CTL, 16 cells in 10 slices; *Grin1* KO, 24 cells in 15 slices; WT CTL, 26 cells in 13 slices. Kruskal-Wallis test followed by Tukey's honest significant difference criterion for multi-group comparison was used in A and B. **C and D:** Representative traces of mEPSC (C) and mIPSC (D) from different experimental groups. mEPSC and mIPSC recordings were done at room temperature in the presence of TTX.

Supplemental tables

Table S1. Effect size r for plots in Figure 1.

Figure 1F (left)	Total # of locations			
P6-7 vs P12-14	0.381			
	Laminar distribution			
Figure 1F (right)	Bin1	Bin2	Bin3	Bin4	Bin5
P6-7 vs P12-14	0.056	0.167	0.343	0.686	0.453
Figure 1G (left)	EPSC amplitude				
P6-7 vs P12-14	0.227				
	Laminar distribution				
Figure 1G (right)	Bin1	Bin2	Bin3	Bin4	Bin5
P6-7 vs P12-14	0.168	0.116	0.184	0.451	0.616
Figure 1H (left)	EPSC charge				
P6-7 vs P12-14	0.253				
	Laminar distribution				
Figure 1H (right)	Bin1	Bin2	Bin3	Bin4	Bin5
P6-7 vs P12-14	0.059	0.107	0.689	0.523	0.577

Table S2. Effect size r for plots in Figure 2-4.

Figure 2H	Response failure rate	Figure 3E	EPSC ratio
-70 mV vs 40 mV	0.351	*Grin1*^{flox} CTL vs *Grin1* KO	0.814

	Active locations		Distance to soma	
Figure 4AB	Upper layers	Lower layers	Upper layers	Lower layers
Grin1 KO slice vs WT CTL slice	0.123	0.056	0.048	0.071
	# of AP		1st AP latency	
Figure 4CD	Upper layers	Lower layers	Upper layers	Lower layers
Grin1 KO slice vs WT CTL slice	0.057	0.342	0.381	0.136

Table S3. Effect size r for plots in Figure 5-6.

Figure 5C, 6A, 6B and 5E	Total # of locations	EPSC amplitude	EPSC charge	Map correlation
Grin1^{flox} CTL vs *Grin1* KO	0.07	0.048	0.132	0.184
Grin1 KO vs WT CTL	0.133	0.131	0.252	0.268
Grin1^{flox} CTL vs WT CTL	0.257	0.121	0.011	0.058

	Laminar distribution of input				
Figure 5C (right)	Bin1	Bin2	Bin3	Bin4	Bin5
Grin1^{flox} CTL vs *Grin1* KO	0.011	0.128	0.033	0.065	0.203
Grin1 KO vs WT CTL	0.119	0.184	0.05	0.021	0.133
Grin1^{flox} CTL vs WT CTL	0.154	0.063	0.063	0.117	0.331
	Laminar distribution EPSC peak amplitude				
Figure 6C (left)	Bin1	Bin2	Bin3	Bin4	Bin5
Grin1^{flox} CTL vs *Grin1* KO	0.124	0.031	0.098	0.319	0.295
Grin1 KO vs WT CTL	0.109	0.103	0.27	0.328	0.189
Grin1^{flox} CTL vs WT CTL	0.039	0.103	0.049	0.043	0.075
	Laminar distribution of EPSC charge				
Figure 6C (right)	Bin1	Bin2	Bin3	Bin4	Bin5
Grin1^{flox} CTL vs *Grin1* KO	0.185	0.135	0.316	0.105	0.095
Grin1 KO vs WT CTL	0.051	0.21	0.17	0.089	0.163
Grin1^{flox} CTL vs WT CTL	0.133	0.022	0.166	0.034	0.253

Table S4. Effect size r for plots in Figure 7.

Figure 7C, 7E, 7F and 7D	Total # of locations	IPSC amplitude	IPSC charge	Map correlation
Grin1^{flox} CTL vs *Grin1* KO	0.215	0.051	0.045	0.059
Grin1 KO vs WT CTL	0.439	0.239	0.389	0.044
Grin1^{flox} CTL vs WT CTL	0.535	0.268	0.405	0.025

Figure 7G (left)	Laminar distribution of input				
	Bin1	Bin2	Bin3	Bin4	Bin5
Grin1^{flox} CTL vs *Grin1* KO	0.143	0.143	0.098	0.245	0.278
Grin1 KO vs WT CTL	0.234	0.14	0.293	0.495	0.402
Grin1^{flox} CTL vs WT CTL	0.344	0.251	0.36	0.614	0.56
**Figure 7G (middle)**	Laminar distribution of IPSC peak amplitude				
	Bin1	Bin2	Bin3	Bin4	Bin5
Grin1^{flox} CTL vs *Grin1* KO	0.122	0.108	0.02	0.104	0.337
Grin1 KO vs WT CTL	0.216	0.176	0.392	0.409	0.248
Grin1^{flox} CTL vs WT CTL	0.266	0.238	0.259	0.441	0.508
**Figure 7G (right)**	Laminar distribution of IPSC charge				
	Bin1	Bin2	Bin3	Bin4	Bin5
Grin1^{flox} CTL vs *Grin1* KO	0.177	0.086	0	0.117	0.306
Grin1 KO vs WT CTL	0.173	0.176	0.416	0.467	0.429
Grin1^{flox} CTL vs WT CTL	0.328	0.253	0.369	0.459	0.568

Table S5. Effect size r for plots in Figure 8.

Figure 8A	E/I index
Grin1^{flox} CTL vs *Grin1* KO	0.111
Grin1 KO vs WT CTL	0.246
Grin1^{flox} CTL vs WT CTL	0.314

Figure 8B	Laminar distribution				
	Bin1	Bin2	Bin3	Bin4	Bin5
Grin1^{flox} CTL vs *Grin1* KO	0.096	0.085	0.14	0.081	0.015
Grin1 KO vs WT CTL	0.071	0.043	0.15	0.394	0.338

Grin1^{flox} CTL vs WT CTL	0.054	0.006	0.038	0.442	0.283

Table S6. Effect size r for plots in Figure 9.

Figure 9A	Local # locations	Local IPSC amplitude	Local IPSC charge
Grin1^{flox} CTL vs *Grin1* KO	0.035	0.216	0.212
Grin1 KO vs WT CTL	0.041	0.227	0.416
Grin1^{flox} CTL vs WT CTL	0.114	0.058	0.306

Figure 9B and 9C	Distal # locations	Distal IPSC amplitude	Distal IPSC charge	Distal E/I index
Grin1^{flox} CTL vs *Grin1* KO	0.028	0.172	0.138	0.05
Grin1 KO vs WT CTL	0.534	0.479	0.512	0.295
Grin1^{flox} CTL vs WT CTL	0.434	0.25	0.37	0.214

	Distribution of distal input				
Figure 9D (left)	Bin1	Bin2	Bin3	Bin4	Bin5
Grin1^{flox} CTL vs *Grin1* KO	0.19	0.173	0.09	0.066	0.11
Grin1 KO vs WT CTL	0.302	0.323	0.254	0.493	0.363
Grin1^{flox} CTL vs WT CTL	0.206	0.154	0.148	0.5	0.438

	Distribution of distal IPSC peak amplitude				
Figure 9D (middle)	Bin1	Bin2	Bin3	Bin4	Bin5
Grin1^{flox} CTL vs *Grin1* KO	0.135	0.155	0.111	0.177	0.098
Grin1 KO vs WT CTL	0.289	0.353	0.559	0.376	0.189
Grin1^{flox} CTL vs WT CTL	0.127	0.13	0.398	0.19	0.285

	Distribution of distal IPSC charge				
Figure 9D (right)	Bin1	Bin2	Bin3	Bin4	Bin5
Grin1^{flox} CTL vs *Grin1* KO	0.053	0.19	0.234	0.129	0.057
Grin1 KO vs WT CTL	0.256	0.35	0.537	0.35	0.355
Grin1^{flox} CTL vs WT CTL	0.219	0.122	0.342	0.248	0.376

Figure 9E	Distribution of distal E/I index				
	Bin1	Bin2	Bin3	Bin4	Bin5
Grin1^{flox} CTL vs *Grin1* KO	0.194	0	0.066	0.024	0.021
Grin1 KO vs WT CTL	0.126	0.128	0.223	0.354	0.328
Grin1^{flox} CTL vs WT CTL	0.006	0.168	0.192	0.34	0.288

Table S7. Effect size r for plots in Figure 9.

Figure 10A-C	mEPSC		
	Frequency	Peak amplitude	Decay tau
Grin1^{flox} CTL vs *Grin1* KO	0.378	0.378	0.378
Grin1 KO vs WT CTL	0.046	0.206	0.161
Grin1^{flox} CTL vs WT CTL	0.24	0.116	0.355
Figure 10D-F	mIPSC		
	Frequency	Peak amplitude	Decay tau
Grin1^{flox} CTL vs *Grin1* KO	0.686	0.461	0.248
Grin1 KO vs WT CTL	0.703	0.642	0.436
Grin1^{flox} CTL vs WT CTL	0.125	0.382	0.125

Figure 10G-H	E/I index	
	Based on Frequency	Based on peak amplitude
Grin1^{flox} CTL vs *Grin1* KO	0.686	0.509
Grin1 KO vs WT CTL	0.714	0.624
Grin1^{flox} CTL vs WT CTL	0.367	0.319
